# Classifying Effluxable Versus Non-Effluxable Compounds Using a Permeability Threshold Based on Fundamental Energy Constraints

**DOI:** 10.3390/pharmaceutics17111455

**Published:** 2025-11-11

**Authors:** Soné Kotze, Kai-Uwe Goss, Andrea Ebert

**Affiliations:** 1 Department of Computational Biology and Chemistry, Helmholtz Centre for Environmental Research (UFZ), Permoserstraße 15, 04318 Leipzig, Germany; 2 Institute of Chemistry, University of Halle-Wittenberg, Kurt-Mothes-Straße 2, 06120 Halle, Germany

**Keywords:** passive permeability, P-glycoprotein, active transport, efflux ratio, energy limit, MDCK

## Abstract

**Background/Objectives**: Predicting whether a compound is subject to active transport is crucial in drug development. We propose a simple threshold for passive membrane permeability (P_m_), derived from the cell’s energy limitation, to identify compounds unlikely to be actively effluxed. **Results**: By considering fundamental cellular energy constraints, our approach provides a mechanistic rationale for why compounds with very high passive permeability in combination with low applied concentration will not undergo active efflux. This moves beyond the empirical observation (such as in previous systems that associate fast-permeating, poorly soluble compounds with low transporter activity) by grounding the prediction in the cell’s energetic limitations. For MDCK (Madin–Darby canine kidney) cells, this threshold—normalized to the applied compound concentration (C_ext_)—was determined to be P_m_×C_ext_ = 10^−1.7^ cm/s×µM. **Methods**: To derive this threshold, we conducted an extensive analysis of literature-reported efflux ratios (ERs) in MDCKII cells overexpressing efflux transporters (MDR1, BCRP, MRP2; 294 datapoints across 136 unique compounds). Concentration-dependent measurements for Amprenavir, Eletriptan, Loperamide, and Quinidine—chosen because these borderline compounds exhibited the highest P_m_×C_ext_ while still being actively effluxed—enabled the most accurate determination of the threshold. Literature ER values were re-evaluated through the experimental determination of reliable P_m_ values, as well as newly measured ER values with MDCK efflux assays. **Conclusions**: The results of these assays and the re-evaluation allowed us to reclassify all but three outliers (compounds with ER > 2.5 and log(P_m_×C_ext_) > −1.7). In contrast, more than 60% of the compounds analyzed without significant ER values (123 compounds) fell above the threshold, in strong agreement with our theory of an energy limitation to active transport. This permeability threshold thus provides a simple and broadly applicable criterion to identify compounds for which active efflux is energetically not feasible and may serve as a practical tool for early drug discovery and optimization, pending further validation in practical applications.

## 1. Introduction

Transport across biological membranes significantly influences the pharmacokinetic and toxicokinetic properties of drugs. Understanding active transport—especially efflux mechanisms—is essential, as these processes greatly impact a compound’s bioavailability, toxicity, and therapeutic efficacy [1]. Active transport of molecules through cell membranes functions in parallel with passive diffusion; however, instead of being driven by concentration gradients, active transport is energy-dependent and can occur against such gradients. As passive membrane permeability increases for more hydrophobic chemicals, more energy is needed for their active transport to counteract their rate of passive diffusion. Given that cells have a limited energy budget, this work sets out to prove the existence of a membrane permeability threshold beyond which active transport of molecules is no longer energetically viable.

Efflux proteins located in the apical membrane such as P-glycoprotein/multi-drug resistant protein 1 (P-gp, MDR1), breast cancer resistance protein (BCRP) and multi-drug resistance associated protein 2 (MRP2) actively pump out foreign substances from cells, which may affect the efficacy of drug uptake [2,3]. Due to their role in decreasing the intracellular concentration of chemotherapeutic agents, the over-expression of such efflux proteins plays a significant role in the resistance of cancerous tissue to multiple drugs [4,5]. Given that P-gp overexpression is linked to multidrug-resistance (MDR)—which diminishes the efficacy of chemotherapeutics, antibiotics, and antivirals—there is a significant need for cost-effective, rapid methods to detect P-gp substrates. These methods could not only assist in predicting a compound’s toxicity, bioavailability, and drug–drug interaction potential [6] but also enable the early exclusion of problematic candidates, thereby mitigating MDR-related challenges during drug discovery [7]. Therefore, it is of great importance in the pharmaceutical industry to ascertain whether drugs are affected by such efflux transporters. Traditionally, the passive membrane permeability (P_m_) of a compound, as well as the involvement of efflux transporters—quantified using a metric known as the efflux ratio (ER)—is determined through bidirectional transport assays using epithelial cell lines such as MDCK or Caco-2. These assays have also been utilized to gather information on the chemical nature of efflux transporter substrates [8,9], in the hopes of enabling the development of predictive in silico models linking physicochemical properties to carrier-mediated efflux (i.e., QSAR models [10], machine learning [11,12]). However, the utility of such models is hindered by their limited interpretability, narrow chemical coverage due to limitation in the training datasets, as well as their reliance on inconsistent datasets.

Efforts have also been made to develop simple and fast interpretations of the molecular characteristics that define efflux substrates [13,14,15,16,17]. These studies seek to establish simple and broadly applicable “rules-of-thumb” for hit-to-lead optimization. It has been proposed that such simple rules might have a more significant impact compared to complex predictive models [6], although their limitations are well recognized, and the need for mechanism-based approaches has been emphasized [18,19]. In this study, we propose another simple and easily applicable, yet mechanistically based, rule-of-thumb: compounds exceeding a certain passive membrane permeability threshold are less likely to exhibit significant transporter-facilitated efflux. The objective of this work was to test this hypothesis and determine whether it could be translated into a guideline for the rapid separation of compounds that could exhibit significant transporter-facilitated efflux from those that cannot, based on their membrane permeability. The permeability-based separation proposed here does not differentiate between efflux substrates and non-substrates. Instead, it differentiates compounds whose ADME characteristics can be significantly affected by efflux transporters from those that cannot, irrespective of whether they are substrates. Establishing a permeability-based separation guideline could prove valuable for drug classification, or could assist in the rational direction of lead optimization towards desired efflux effects. Since it was shown recently by Dahley et al. [20] that the biological Caco-2/MDCK membrane permeability of drugs is well predicted by the solubility diffusion model (SDM), permeability-based separation can in most cases be performed without the need for experimental assays. Furthermore, because this rule is based on a fundamental physical principle (the energy limit of the cell) it is not limited to P-gp, but should also apply to any efflux transporter that consumes ATP.

The aims of this study were to: (i) evaluate existing MDCKII ER data for MDR1, BCRP and MRP2, and establish a crude membrane permeability cut-off above which compounds appear to not be affected by active efflux. Compounds that seem to contradict this threshold can then be flagged as outliers and subsequently examined. Secondly, it was aimed (ii) to identify compounds located at or near the threshold for further investigation with concentration-dependent monolayer efflux assays. The resulting data can then be used to quantify the maximal flux rate (i.e., the maximum amount of compound that can be actively effluxed per unit time) and the corresponding energy limit. Finally, we aimed (iii) to link this energy limit with a specific P_m_ value and perform a sensitivity analysis to highlight the caveats and limitations associated with defining a rule-of-thumb P_m_ threshold.

## 2. Theory

### 2.1. Transport Model

**Figure 1:**
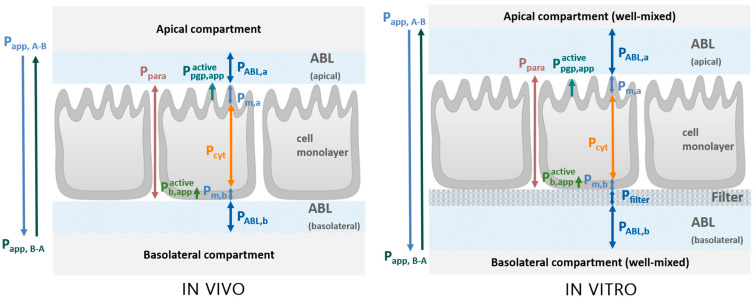
**Schematic representation of permeation barriers in vivo and in vitro.** In vivo (**left**), well-mixed donor and acceptor compartments are separated by the apical ABL, the cell monolayer, and the basolateral ABL. The in vitro system (**right**) additionally includes a filter layer and exhibits a thicker ABL (not to scale). Reproduced from Kotze et al. [21], licensed under CC BY 4.0. In the case of in vivo transport in intestinal cells for example, the apical compartment is the lumen of the gastrointestinal tract (GIT), and the basolateral compartment is the blood.

As compounds permeate from one compartment to another across a cell layer, they encounter various resistances, both in series and in parallel. This includes aqueous boundary layers (ABL), as well as the cell layer itself, consisting of the basolateral and apical membranes, as well as the cytosol. The permeability (P) of each barrier is inversely proportional to its resistance. As is evident in Figure 1, the unidirectional active transport facilitated by P-gp (denoted as $P_{pgp,app}^{active}$) and a basolateral uptake transporter (denoted as $P_{b,app}^{active}$) occurs in parallel to the passive diffusion through the apical membrane (P_m,a_) and the basolateral membrane (P_m,b_), respectively. The in vitro situation during MDCK/Caco-2 assays is comparable to the in vivo scenario, save for the presence of a permeable filter on which cells are grown, as well as thicker unstirred water layers. A detailed description of the conceptual model used, as well as the various resistances and how they can be quantified, can be found in Kotze et al. [21].

The total permeability through all barriers (as measured by transport assays) is referred to as the apparent permeability (P_app_). Consequently, P_app_, as a function of all individual permeabilities, can be broken down into the contributions from each component, enabling the separate evaluation and quantification of permeability through each layer (see Equations (S1) to (S8) in the Supplementary Materials S1 (Section S1) for modeled permeabilities and local compound concentrations as described in detail in Kotze et al. [21]).

### 2.2. Maximal Active Transport Flux and Borderline Compounds

It is crucial to distinguish between the two different threshold values discussed in this work: the maximal flux and the associated maximal passive membrane permeability. The maximal flux, or J_pgp,active_, refers to the maximum moles of compound that can be actively transported by any transporter per unit area and time (μmol/cm^2^/s) assuming the cell has limits to the energy it can invest in its transporters. The energy limit thus corresponds to the maximal J_pgp,active_ possible given limited energy production of the cell. Both the value for the active efflux permeability $P_{pgp,app}^{active}$ (cm/s) facilitated by P-gp from the apical membrane as well as the actively transported flux J_pgp,active_ at a given apical freely dissolved concentration can be extracted from model fits of experimental MDCK data. It is important to consider only the freely dissolved concentration because only this fraction can permeate the membrane. A theoretical calculation of the maximal possible J_pgp,active_ based on the cell’s ATP turnover can also be performed to compare with the empirical value as a plausibility check; see Supplementary Materials S1 (Section S2). However, J_pgp,active_ is an impractical metric, since it is complex to determine and not meaningful for the average scientist. Thus, we aimed to ultimately link it with passive membrane permeability, P_m_, which is usually readily available, or easily determinable if not. The P_m_ threshold is thus the specific passive membrane permeability value where the rate of passive influx of a chemical would require an active efflux rate that exceeds the cell’s maximal J_pgp,active_.

As such, chemicals with a P_m_ value above the threshold would be unlikely to show significant efflux, since their high permeability would impose an unsustainable energy demand on the cell. The link between J_pgp,active_ and P_m_ arises from the assumption that almost all the compound passively diffusing into the cells is actively effluxed, which is a valid approximation for high efflux ratios. If membrane permeability poses the main passive resistance, passive influx corresponds to P_m_×C_ext_. For significant ER values, the energy limit can thus be approximated by the applied concentration-corrected membrane permeability, P_m_×C_ext_. Although concentrations in MDCK/Caco-2 efflux assays typically vary only within a narrow range (1–100 µM), therapeutic plasma concentrations span more than six orders of magnitude [22]. It is therefore essential to take C_ext_ into account when defining the threshold.

Since J_pgp,active_ depends on several factors, the resultant P_m_×C_ext_ threshold would not be a definite cut-off line. Rather, one could expect the existence of a transitional range of permeability values that separate compounds that cannot be effectively effluxed from those that can. For compounds that have a P_m_×C_ext_ value within this window (which we designate as “borderline compounds”) concentration plays a particularly pivotal role. For such borderline compounds, we observed concentration-dependent saturation of active transport. The rationale is that at low concentrations, compounds can be efficiently effluxed without substantially straining the cell’s energy budget. However, as the concentration increases, a larger quantity of the compound enters the cell, which brings the increased efflux closer to reaching the energy limit. Finally, at a certain higher concentration and beyond, the rate of passive permeation into the cell far exceeds the rate at which it can be effluxed, due to the depletion of the energy budget.

## 3. Material and Methods

### 3.1. Determination of Passive Membrane Permeability P_m_

To determine the energy limit, a reliable P_m_ is needed. P_m_ can be determined experimentally, or predicted with in silico methods. The pH-partition hypothesis dictates that only the neutral fraction (f_n_) of a chemical can traverse membranes by passive diffusion, because the permeability of the ionic fraction is negligible in comparison [23]. The P_m_ of ionizable chemicals is therefore dependent on pH, or the neutral fraction f_n_:


(1) $$P_{m}=P_{0}\times f_{n}$$

The interconversion between the intrinsic permeability P_0_ and P_m_ is straightforward when the neutral fraction is known. For all compounds in this study, pKa values were used to determine f_n_ at pH 7.4 according to Escher et al. [24]. Experimental pKa values were preferred; however, where no experimental pKa could be sourced, pKa values were instead determined using the software ACD/pKa GALAS from ACD/Percepta (version 2020.1.2, Advanced Chemistry Development, Inc. (ACD/Labs), Toronto, ON, Canada, www.acdlabs.com, accessed on 1 September 2025) [25]. For zwitterions, only the non-charged neutral species was assumed to permeate the membrane, and the respective fraction was calculated according to Ebert and Dahley [26]. See Tables S1–S3 in the Supplementary Materials S2 for details.

For epithelial cells such as the MDCK line used in this study, it is crucial to note that microvilli are created by the folded apical membrane. Consequently, it is believed to have a greater surface area compared to the basolateral membrane. If a difference in surface area indeed exists between the apical and basolateral membrane, it is suspected to have a significant effect on the resulting energy limit of this work. In our previous study we speculated that the factor of 24 [27] we routinely implemented to account for the greater apical membrane surface area was likely excessive [21]. As such, in the main text of this study we used a more conservative apical membrane surface area factor (SA) of 7.5 (determined specifically for MDCKII cells as the average value of these cells cultivated on different filters [28]), and compared this to the two other proposed factors of 24 and 1 on the extreme ends of the probable range in the Supplementary Materials S1 (Sections S9–S16). The factor of 1 naturally indicates that the surface areas between apical and basolateral membranes are identical.

#### 3.1.1. In Silico Prediction of P_m_ Using UFZ-LSERD/COSMO-RS

Experimental P_m_ values are the most reliable, so for this work such values were preferred when available for a given compound. When no reliably extracted experimental P_0_ value was available for the calculation of P_m_, P_0_ was estimated based on the solubility-diffusion model (SDM) by utilizing hexadecane as a model for the hydrocarbon core of the membrane [20,29]:

(2) $$P_{0,SDM}=\frac{D_{hex}\times K_{hex/w}}{x_{m}}$$

where $K_{hex/w}$ is the hexadecane–water partition coefficient of the compound, $D_{hex}$ is the diffusion coefficient of the compound in hexadecane, and $x_{m}$ refers to the thickness of the hexadecane-like hydrocarbon core of the cell membrane. It is assumed that $D_{hex}$ is one-tenth of $D_{w}$ [29], and thus it was calculated based on the molecular weight of the compound, adjusted for the temperature of 37 °C (see Supplementary Materials S1 (Section S1)). The $K_{hex/w}$ was obtained from the LSER database [30]. $K_{hex/w}$ values determined from experimental descriptors were given precedence and duly categorized. In cases where experimental descriptors were not available, $K_{hex/w}$ values derived from calculated descriptors were utilized instead. For comparative purposes, $K_{hex/w}$ values were also generated for all compounds using the quantum chemically based software COSMOtherm (version C30, release 18, COSMOlogic GmbH & Co. KG, Leverkusen, Germany, www.cosmologic.de, accessed on 30 September 2021) [31]. COSMOtherm values were only used for zwitterions, and compounds with a molecular weight > 1000 g/mol. The P_0_ of the MDCK cell membrane P_0,MDCK_ was subsequently calculated from P_0,SDM_ based on the empirical correlation established by Dahley et al. [20]:


(3) $$P_{0,MDCK}=0.84\;logP_{0,SDM}-1.85$$

#### 3.1.2. Experimental Determination of P_m_ with Bidirectional MDCK Assays

Membrane permeability P_m_ and the resultant P_0_ values can also be determined with in vitro bidirectional assays. In this study, P_0_ values for all compounds were preferentially sourced from our own experiments and from Ebert et al. [32], who extracted P_0_ values from experimental Caco-2/MDCK P_app_ data from several sources while accounting for the many pitfalls associated with P_0_ determination. Consequently, they are regarded as the most dependable P_0_ values available.

#### 3.1.3. Experimental Determination of P_m_ with PAMPA and SDM

Using hexadecane as the membrane in HDM-PAMPA (Parallel Artificial Membrane Permeability Assay) has been shown an effective way to determine reliable experimental K_hex/w_ [33]. These $K_{hex/w}\;$ values could subsequently be applied via Equations (2) and (3) to calculate a P_m_ for MDCK cells that is more reliable than those generated from predicted $K_{hex/w}\;$ values [20]. For this work, HDM-PAMPA assays were performed for some compounds at suitable pH values. Detailed descriptions of the methodology used can be found in an accompanying study by Dahley et al. [33]. Further details about the experiments first reported in Dahley et al. [33] that are relevant to this study (including the chemicals and reagents used, the experimental conditions, the experimentally obtained P_app_, P_0_ and K_hex/w_ values, as well as the resultant P_m_ values calculated from it, etc.) can be found in the Supplementary Materials S1 (Sections S3 and S4) and S2 (Tables S5 and S6).

### 3.2. Analysis of ER Data

#### 3.2.1. Evaluation of ER Data from Literature

Three distinct datasets of ER values were compiled for substrates of P-gp, BCRP and MRP2, as these are the three most clinically relevant apically located efflux transporters. A literature search was conducted to identify compounds with an ER ≥ 2.5 [34], measured using bidirectional MDCK transport assays. For compounds with ER values reported by multiple sources, all values were included in the analyses, even if some sources reported conflicting ER values below the threshold. A total of 294 datapoints were collected, representing 136 unique compounds, with the majority of the data coming from MDCKII-MDR1 assays, since assays for MDCK-BCRP and MRP2 are considerably less common. We note that this compilation is not exhaustive. MDCKII-MDR1 assay data from 24 different sources were used [34,35,36,37,38,39,40,41,42,43,44,45,46,47,48,49,50,51,52,53,54,55,56,57]. The dataset included 227 data points (i.e., ER values) from 107 distinct compounds. MDCK-BCRP assay data was obtained from 12 different sources [56,58,59,60,61,62,63,64,65,66,67,68]. The dataset contained 52 data points from 33 distinct compounds. MDCK-MRP2 assay data was obtained from 8 different sources [69,70,71,72,73,74,75,76]. The dataset contained 15 data points from 11 distinct compounds. A fourth, separate dataset was also curated for compounds with insignificant ER values below the cut-off line. For this, data was obtained from 4 sources [36,38,39,40]. This dataset comprised 125 datapoints representing 123 compounds. For all datasets, compounds with a permanent charge were excluded from subsequent analyses because they lack a neutral fraction capable of passively permeating through the hydrophobic core of the membrane.

**Figure 2:**
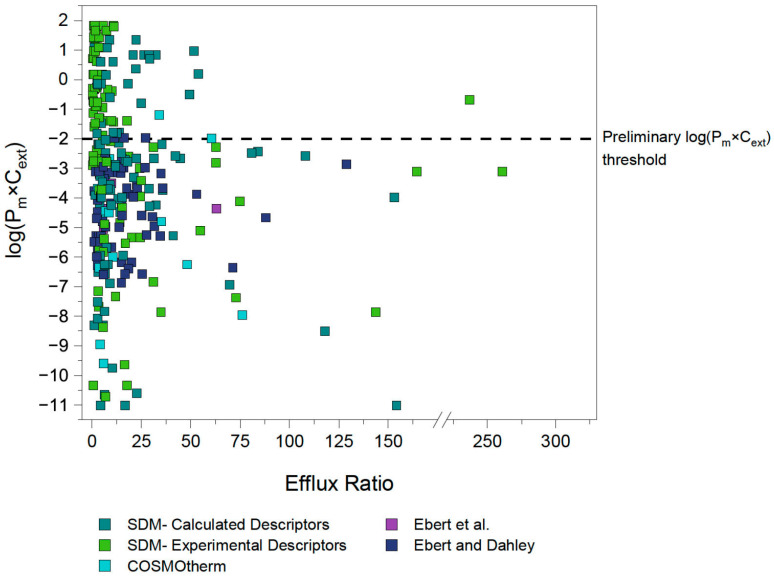
**ER and log(P_m_×C) values of compounds shown to have significant (>2.5) ER values as determined with MDCK assays.** All ER values sourced from literature for MDCKII-MDR1, -BCRP and -MRP2 bidirectional assays. Experimental P_m_ values sourced from Ebert et al. [32] and Ebert and Dahley [26] were preferred. If no experimental P_m_ values were available, P_m_ was calculated with the Solubility Diffusion Model (SDM) using K_hex/w_ values determined preferably from experimental descriptors if available, and calculated descriptors if not. For zwitterions and compounds with MW > 1000 g/mol, COSMOtherm was used to calculate P_m_.

The P_m_ for each compound was then calculated using Equation (1) and the corresponding applied concentrations used by the respective studies were noted and used to calculate the concentration-corrected P_m_ values (P_m_×C_ext_). The MDCK ER values from the studies of all three transporters and the P_m_×C_ext_ values determined for each respective compound are depicted in Figure 2. Tabulated values of Pgp/MDR1, BCRP and MRP2 substrates along with their chemical properties can be found in the Supplementary Materials 2 (Tables S1, S2 and S3, respectively), along with the concentration-corrected P_m_ values (Table S4).

#### 3.2.2. Re-Determining the ER of Outlier Compounds with Bidirectional MDCK Assays

##### Chemicals and Reagents

All chemicals and reagents can be found in the Supplementary Materials S1 (Section S3).

##### Cells and Cell Culture

MDCKII-MDR1 cells were obtained from The Netherlands Cancer Institute (Amsterdam, The Netherlands). The cell medium was Dulbecco’s modified Eagle medium (DMEM) (1X) + GlutaMAX™-I supplemented with 10% fetal bovine serum (FBS), 100 U/mL penicillin and 100 µg/mL streptomycin. Cells were maintained at 37 °C in an atmosphere of 5% CO_2_ and passaged twice a week.

##### Bidirectional MDCK Assays

MDCK bidirectional transport assays were performed as described in Kotze et al. [21,77]. MDCKII-MDR1 cells (passages 20–40) were seeded at a density of 1.5 × 10^5^ cells/insert onto 12-well transwell inserts (CellQART, Northeim, Germany; pore size: 0.4 µm; filter thickness: 11.5 µm, porosity: 100 × 10^6^ pores/cm^2^). After seeding, cells were maintained as outlined in Cells and Cell Culture Section and grown for 4 days. The day prior to the experiment, the cell medium was refreshed. The transport buffer was HBSS and HEPES with a pH of 7.4. Stock solutions were prepared in the transport buffer. Prior to the experiment, the pH of the stock solutions and buffer was controlled with a Rapid_pH™ Automated pH Meter (Hudson Robotics, Inc., Springfield, NJ, USA), as was the pH of all samples after the conclusion of the experiment. Inserts were placed in 12-well plates from TPP Techno Plastic Products AG (Trasadingen, Switzerland). Sampling was conducted at 3 to 4 time intervals, with the duration tailored specifically for each compound and measurement direction to guarantee sink conditions. In between sampling steps, plates were placed in a Titramax and Inkubator 1000 orbital shaking incubator from Heidolph Instruments GmbH & Co. (Schwabach, Germany) at 450 rpm and 37 °C. At the final sampling step, the donor compartment was sampled for mass balance calculations. For each insert, the trans-epithelial electrical resistance (TEER) across the cell monolayer was measured at 37 °C using an EVOM epithelial tissue volt/ohmmeter (World Precision Instruments Inc., Sarasota, FL, USA) before and after the experiment to ensure that cell monolayer remained intact throughout the experiment. Lucifer yellow (LY) was utilized as a paracellular marker to further confirm the integrity of the cell monolayer. For this, the LY permeability of each insert was assessed post-experiment by measuring the fluorescence intensity (excitation: 485 nm, emission: 538 nm) in samples from the basolateral compartment using a SpectraMAX Gemini EM spectrophotometer (Molecular Devices LLC., San Jose, CA, USA). Inserts that demonstrated a LY permeability greater than the pre-defined threshold of 1.5 × 10^6^ cm/s were excluded [78], unless their P_app_ results were qualitatively consistent with those of replicate inserts that fell within the LY threshold.

Samples were analyzed with an Infinity II 1260 LC system coupled with a 6420 triple quadrupole 145 with ESI source (Agilent Technologies Inc., Santa Clara, CA, USA). Either a Kinetex^®^ F5 (2.6 µm; 100 Å; 50 × 3.0 mm) or C18 (2.6 µm; 100 Å; 50 × 3.0 mm) LC column was used (Phenomenex Inc., Torrance, CA, USA). P_app_ was calculated from acceptor compartment concentrations as described in Kotze et al. [21]. P_app_ values at each time point were corrected using the calculated recovery, as done by Neuhoff [79]. The data is shown as the mean of the recovery-corrected P_app_ ± standard deviation from at least two samples from each replicate. To account for lag time [80] the first time point in the A ⟶ B direction was excluded. The ER was determined as the ratio of the mean P_app_ values in the B ⟶ A direction and A ⟶ B direction according to:


(4) $$ER\equiv \frac{J_{B⟶A}}{J_{A⟶B}}\equiv \frac{P_{app,B⟶A}}{P_{app,A⟶B}}$$

#### 3.2.3. Concentration-Dependent MDCK Assays for Borderline Compounds

Bidirectional MDCK-MDR1 assays were performed as described in Bidirectional MDCK Assays Section and Kotze et al. [21] to determine P_app_ values as well as the ER using stock solutions with varying concentrations. Concentrations were selected to cover the range of viable concentrations within the boundaries of the experimental protocol and LC-MS quantification. When DMSO was necessary for solubility, the total concentration was maintained at a maximum of 0.1%, and the transport buffer was supplemented with the same concentration to avoid the creation of a DMSO gradient across compartments. MDCK assays conducted for this section were performed in triplicate, with sampling occurring at four consistent time intervals. The data from these assays are presented as the mean of the recovery-corrected P_app_ ± standard deviation from at least three samples from each replicate.

#### 3.2.4. Determination of $P_{pgp,app}^{active}$ and Maximal Flux J_pgp,active_

The recovery-corrected A ⟶ B and B ⟶ A permeabilities determined for each concentration of the assays in Section 3.2.3 were used in the evaluation of the saturation experiments. The thickness of the apical and basolateral ABL, along with the thickness of the filter and its effective surface area, as well as the P_0_, D_w_, D_cyt_ and pKa values for each compound were used to determine the permeability through the individual resistances (ABLs, membranes, cytosol and filter). The paracellular permeability was determined as described by Bittermann and Goss [29], with a factor of 0.1 applied to ensure that its final value matches the paracellular permeabilities generally observed for our MDCKII-MDR1 cell set-up [21]. Based on the compound’s pKa values and the external pH, the external neutral fraction was calculated according to Escher et al. [24]. After determining the cytosolic pH as a function of the external pH according to Dahley et al. [20], the neutral fraction in the cytosol was similarly calculated

The experimental P_app_ and known P_0_ values were subsequently utilized to fit the apparent permeabilities facilitated by P-gp ($P_{pgp,app}^{active}$) and the basolateral uptake transporter $\left(P_{b,app}^{active}\right)$. The fitting of P_app A⟶B_ (see Equation (S3)) was done for each concentration using the Excel SOLVER function which minimized the difference between the calculated and experimental apparent permeability by varying $P_{pgp,app}^{active}$. Using the starting concentrations (μmol L^−1^), as well as the extracted $P_{pgp,app}^{active}$, it was possible to calculate the concentration of the compound adjacent to the apical membrane in the cytosol (C_cyt,a_) (see Equation (S6)). The flux of actively transported compound, J_pgp,active_, was then derived from the product of $P_{pgp,app}^{active}$ and C_cyt,a_ in μmol cm^−2^ s^−1^. Ideally, a sequential calculation would have been performed: First, for the basolateral transporter $P_{b,app}^{active}$, by fitting the B ⟶ A permeability (see Equation (S4)) assuming that the apical membrane does not pose a significant resistance in this transport direction. Subsequently $P_{pgp,app}^{active}$ would be determined by fitting the A ⟶ B permeability. However, due to unexpected experimental effects, only A ⟶ B permeability could be fitted to determine ${PS}_{pgp,app}^{active}$, under the assumption of insignificant basolateral active transport. This is elaborated upon and justified in the Supplementary Materials S1 (Section S7).

#### 3.2.5. Linking the Maximal Flux Value with a Membrane Permeability Threshold

For the compounds that exhibited saturation effects in the concentration-dependent MDCK assays and therefore evidently reached the maximum flux plateau, classic non-linear Michaelis–Menten fits for extracted J_pgp,active_ depending on C_cyt,a_ were conducted with IGOR Pro 7 (WaveMetrics Inc., Lake Oswego, OR, USA) in order to ascertain the maximal J_pgp,active_. The maximal J_pgp,active_ was then used to calculate the corresponding threshold P_m_ value depending on ER (i.e., the highest passive membrane permeability a compound can have in order to potentially be subject to active efflux), again using the Excel solver function to optimize P_m_ for various $P_{pgp,app}^{active}$, minimizing J_pgp,active,max_ − $P_{pgp,app}^{active}$ × C_cyt,a_. ER and C_cyt,a_ were calculated using Equations (S5) and (S6), respectively. In contrast to our earlier assumption that nearly all compounds passively diffusing into the membrane are subsequently effluxed, this approach also allows determination of P_m_ for low ER values, where that assumption is not valid. In essence, P_m_ was back-calculated based on P-gp activity, and in so doing, it was established what P_m_ value is possible for P-gp activity to equal the maximal flux value. Compound concentrations, paracellular transport and the apical membrane surface area were varied for the sensitivity analysis of the P_m_ threshold.

## 4. Results and Discussion

### 4.1. First Estimation of P_m_×C_ext_ Limit

To validate our assumption of a P_m_×C_ext_ limit for active transport, the efflux ratios for P-gp, BCRP and MRP2 substrates were extracted from literature as described in Section 3.2.1. Figure 2 shows the P_m_×C_ext_ plotted against the reported efflux ratios for a total of 286 datapoints, representing 132 unique compounds (after exclusions, see details in Section 4.3). Different colors indicate the source of each P_m_ value, which in turn reflects the reliability of the experimental or predicted data.

A rough approximation for the cut-off value of compound permeability was defined to guide the initial investigations. This cut-off P_m_×C_ext_ value was identified by evaluating the distribution of the data (Figure 2). Data points which had P_m_×C_ext_ values based on the reliable P_0_ values extracted by Ebert et al. [32] and Ebert and Dahley [26] were given particular weight. As a result, a preliminary threshold of log(P_m_×C_ext_) = −2 was set as an initial approximation of where the energy threshold (and therefore the associated membrane permeability threshold) could lie, as it can be observed that none of the reliable experimental P_m_×C_ext_ values reported by Ebert et al. [32] exceed this value.

### 4.2. Identification and Investigation of Outliers

**Figure 3:**
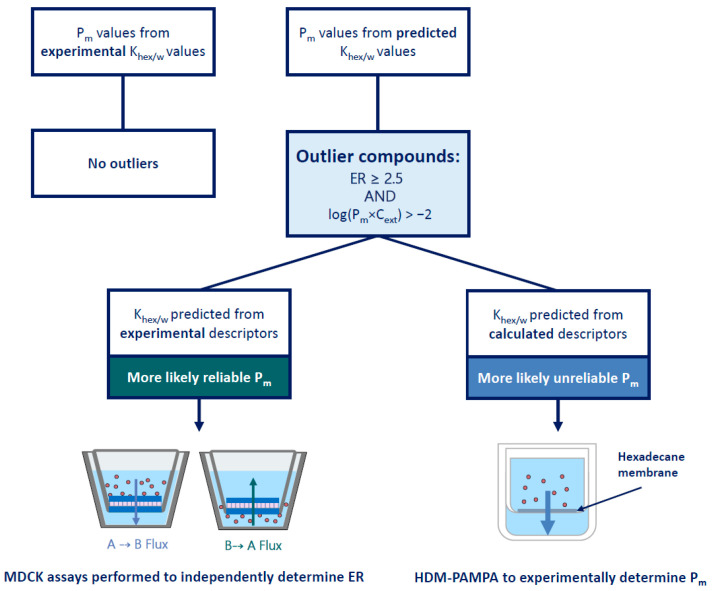
**Schematic depicting the methodical experimental investigation of outlier compounds based on the assumed reliability of P_m_ values.** All outlier compounds had P_m_ values determined from predicted K_hex/w_ values. If the K_hex/w_ was determined from experimental descriptors (reliable P_m_ values), MDCK assays would preferentially be performed to confirm the reported ER. If the K_hex/w_ was determined from calculated descriptors (unreliable P_m_ values), HDM-PAMPA was preferentially performed to determine a reliable P_m_ based on experimental K_hex/w_ values.

All 59 datapoints (41 compounds) with an ER ≥ 2.5 and with a log(P_m_×C_ext_) > −2 were thus identified as tentative outliers to our hypothesis, see Table S6. Owing to the specific threshold selection, all outliers had P_m_ values predicted from $K_{hex/w}\;$ values determined either with experimental or calculated descriptors. $K_{hex/w}\;$ values obtained from experimental descriptors are typically considered as quite reliable, suggesting that it was more likely that the reported ER values for these outliers were incorrect. Therefore, MDCK assays were conducted to determine the ER independently in an effort to replicate the findings of the original study. Only MDCKII-MDR1 assays were performed since only the MDR1 dataset had outliers in this category. In contrast, $K_{hex/w}\;$ values from calculated descriptors are deemed less reliable, so for the corresponding outliers it was assumed that the calculated P_m_ was false. HDM-PAMPA experiments were preferentially performed to obtain more reliable $K_{hex/w}\;$ values as already described in Section 3.1.3. Figure 3 presents a schematic summarizing the process used to determine how each outlier compound was probed. These assumptions served as a general guideline; however, for some compounds, both MDCK as well as HDM-PAMPA were eventually performed.

### 4.3. Outlier Reclassification

**Figure 4:**
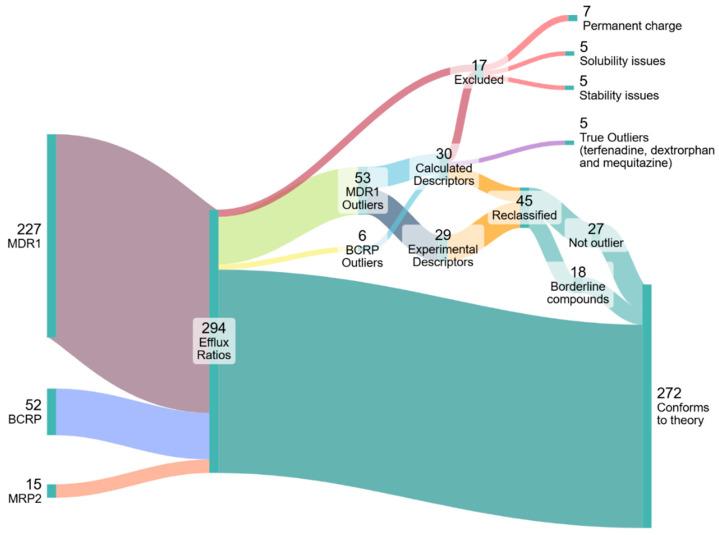
**Diagram illustrating the categorization and analysis of 294 efflux ratios (136 distinct compounds) derived from MDR1, BCRP, and MRP2 transporter datasets.** Certain chemicals were excluded for having a permanent charge, or for exhibiting solubility stability issues. Outliers were identified and investigated with MDCK or HDM-PAMPA assays, or by alternative methods. As is evident, the vast majority of outliers were reclassified and found to conform to the energy limitation theory, as 90% of the data supports the existence of a membrane permeability cut-off for compounds exhibiting significant efflux.

Figure 4 represents the collection, analysis and reclassification of the data. Five outlier compounds (gefitinib, lopinavir, ritonavir, zotepine and dasatinib) were excluded for exhibiting stability and solubility issues that precluded reliable PAMPA/MDCK assays. Notably, two-thirds of the MDR1 outlier compounds originated from just three of the 22 sources. Data obtained from Wager et al. [34] accounted for over 30% of the outliers. Furthermore, with the exception of one compound, every outlier reported by Wager et al. [34] was categorized under “Experimental Descriptors”, suggesting that the ER values (rather than the P_m_) for these outliers are likely questionable. Significant ER values reported by Wager et al. as well as two other sources overrepresented in the outlier data—Obradovic et al. [43] and Wang et al. [81]—were frequently contradicted by other sources that reported no significant ER. As such, the values from these sources were approached with caution. Accordingly, the 22 outliers from these sources were reclassified based on the contradictory non-significant ER values obtained from our own MDCK assays and/or from other sources. Two outliers were reclassified as non-outliers based on newly determined P_m_ values from PAMPA experiments. Ten outliers were reclassified as borderline compounds based on P_m_ values—often newly determined with PAMPA—that placed their log(P_m_×C) values between −2 and −1. The outliers, their reclassification and the basis for it can be found in Table S6 of the Supplementary Materials. The results of the MDCK and PAMPA assays can be found in the Supplementary Materials S1 (Sections S4 and S5) as well as S2 (Table S5).

After evaluation, there are three remaining outliers: dextrorphan, mequitazine and terfenadine. Both dextrorphan and mequitazine have reported ER values (2.6 and 2.8, respectively) that barely surpass the significance threshold and are thus not very convincing outliers. For terfenadine, our own MDCK assays did find a significant, albeit also very low ER value of 2.8. Furthermore, a newly determined PAMPA K_hex/w_ value for terfenadine confirmed a log(P_m_×C_ext_) value above the threshold, at 0.08. However, this compound exhibits very low recovery in the MDCK assays, which likely would have been the situation in assays from other sources as well. These low recovery rates may very well lead to a false ER.

### 4.4. Concentration-Dependent Investigation of Borderline Compounds

**Figure 5:**
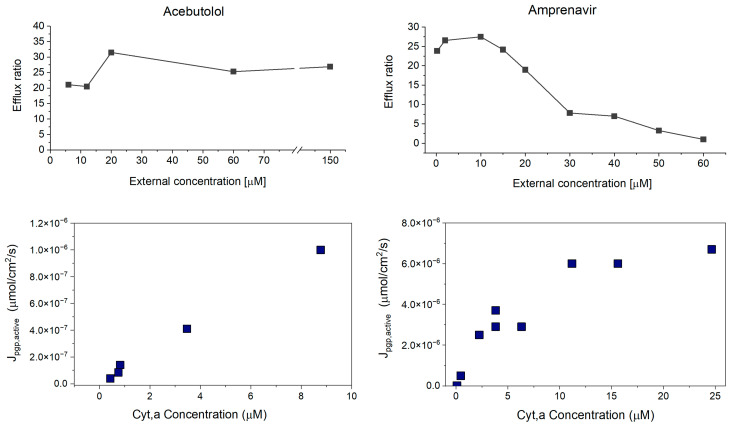
**ER vs. external concentration for the borderline compound amprenavir (top), compared to the low P_m_ compound acebutolol (bottom).** For borderline compounds, the ER decreases with increasing concentration. For the low P_m_ compound, the ER stays constant even at high external concentrations.

Figure 5 shows the results of the concentration-dependence assays for a borderline compound (amprenavir) compared to a low P_m_ compound (acebutolol). The top panels show ER values as a function of applied assay concentration. The bottom panels show the J_pgp,active_ values (μmol/cm^2^/s) calculated from the P_app,A⟶B_ values at each concentration (Section 3.2.4). Similar graphs for all compounds tested can be found in the Supplementary Materials S1 (Sections S6, S10–S12), along with tabulated P_app_, recovery and ER values at each concentration.

For the low P_m_ compound acebutolol, P-gp has not reached saturation, and there is no change in ER values even at very high concentrations. In contrast, for the borderline compound amprenavir, the decline in ER values is attributed to classic saturation effects of the P-gp transporter. This supports our theory that there is concentration-dependent saturation of active transport for compounds near the maximal flux limit. The decline in ER can be attributed to classic saturation effects of the P-gp transporter. At lower concentrations of the compound, the P-gp transporter is not saturated, leading to high transport rates and consequently high ER values. At higher compound concentrations, the transporter has likely reached saturation, and active transport does not further increase with concentration, meaning $P_{pgp,app}^{active}$ decreases. Therefore, passive permeation (permeability × concentration) becomes more dominant as it increases proportionally to concentration, since P_m_ is expected to remain constant.

The observed saturation effects coinciding with the energy plateau are unlikely to be coincidental. Both phenomena result in the same outcome: no further increase in transporter activity with increasing concentration beyond a certain maximum flux value. As such, the energy limit imposed by physical principles is enforced by the cell through the mechanism of saturation. While transporter saturation and its effect on transporter-facilitated efflux have been described previously [82,83,84], this research is, to our knowledge, the first to associate it with an energy threshold. It is important to note that this does not imply that every observed saturation effect is caused by an energetic limitation; rather, a saturation effect is always observed before the energetic limit is reached, which is plausible from an evolutionary perspective, as it avoids the energy depletion of the cell. It is unclear how exactly the saturation effect would be linked to the energy limit. One hypothesis is that ATP binding kinetics may become rate-limiting at high active transport rates; however, this potential relationship is beyond the scope of the present study.

Tachibana et al. [85] previously applied a sophisticated model to extract J_pgp,active,max_ (referred to as V_max_ in their work) from concentration-dependent measurements reported by Shirasaka et al. [86] for verapamil, vinblastine, and quinidine. Their model accounted for passive permeation into and out of the cell on both the apical and basolateral sides of the membrane, as well as unidirectional P-gp transport. While our model for obtaining the K_m_ value differs from theirs, both approaches use the intracellular K_m_ parameter relevant for transporter saturation rather than the extracellular one. The main difference between their approach and ours is that we also included paracellular transport and ABL resistance. It should be emphasized that ABL resistance cannot simply be lumped together with passive permeability; only passive permeability runs in parallel with active transport, whereas the ABL does not. Moreover, paracellular transport can strongly influence P_app_, since P_app_ will not fall below P_para_, even when P_trans_ is negligibly low because of active efflux.

Unfortunately, re-analyzing their published data with our model did not yield reliable results, see Supplementary Materials S1 (Section S7). A fourth compound, digoxin, was included in the study by Shirasaka et al. [86]. It showed no signs of saturation across the tested concentration range (up to 26 µM). With a P_0_ of 4.14 [32], corresponding to a log P_m_×C_ext_ of –2.72, this completely neutral compound lies well below our permeability threshold—fully consistent with our model. Finally, the V_max_ of 5.7 × 10^−6^ µmol/cm^2^/s for quinidine, obtained by Heikkinen et al. [84] using a five-compartment model that accounted for ABL effects, is in good agreement with our own J_pgp,active,max_ of 9.7 × 10^−6^ µmol/cm^2^/s (for SA = 1) derived from our quinidine experiments.

Therefore, it is worth reiterating that the conditions under which the experiments are conducted are critical for obtaining meaningful results in MDCK efflux assays. We have previously demonstrated that ER values can change based on the pH [21], which means that pH can play a role in the misclassification of compounds. In this work we show that using concentrations that are too high can also result in the erroneous conclusion that compounds are not P-gp substrates.

### 4.5. Empirical Determination of the Energy Limit

**Figure 6:**
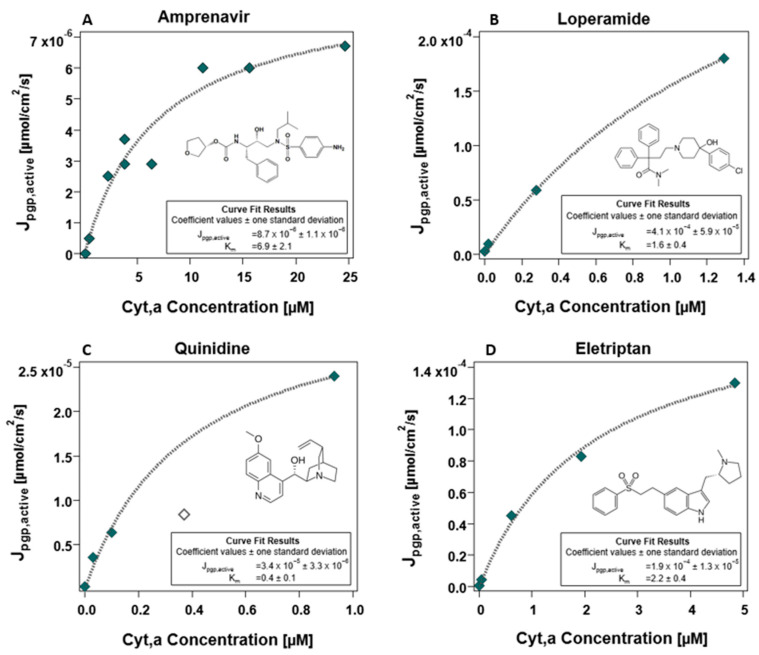
**Non-linear Michaelis–Menten fits for borderline compounds exhibiting the maximal flux (J_pgp,active_) plateau**, **for (A) amprenavir, (B) loperamide, (C) quinidine and (D) eletriptan.** Markers represent the concentration-dependent calculated J_pgp,active_ values, which are derived from fitted P-gp activity values obtained from MDCK assays. For the compound quinidine (**C**), the unfilled marker was not included in the fit.

Four out of the six compounds suspected to be borderline compounds (for which concentration-dependent assays were performed) exhibited the expected plateau when the transport of these compounds started reaching the maximal J_pgp,active_. Due to the observation of saturation effects with these compounds which evidently reached the maximal flux plateau, Michaelis–Menten fits were conducted as described in Section 3.2.5, the results of which are depicted in Figure 6. The final J_pgp,active,max_ value was determined from the average of the individual maximum flux values determined for the four compounds depicted in Figure 6. Analogous fits for the same compounds using the other apical membrane surface area factors of 1 and 24 as well as the resultant mean J_pgp,active_ values in these cases can be found in the Supplementary Materials S1 (Sections S13–S15). The mean (N = 4) J_pgp,active_ determined for the case considered as most plausible (apical membrane surface area factor of 7.5) was determined to be 1.6 × 10^−4^ μmol/cm^2^/s (range: 8.7 × 10^−6^ to 4.4 × 10^−4^ μmol/cm^2^/s). This value is quite similar to a rough estimate of 7.4 × 10^−5^ μmol/cm^2^/s obtained from mechanistic energy constraint considerations outlined in S1 (Section S2).

Since the maximal flux value is based on cellular energy constraints, we expect that all borderline compounds should plateau at the same maximal J_pgp,active_. As is evident from Figure 6, this is not quite the case. This is easily explained when one considers the influence of P_0_ values in determining J_pgp,active_. P_0_ values always come with some threshold of error, and the source of the P_0_ is the biggest determining factor of the level of error. Quinidine has a very reliable experimental P_0_ value determined from MDCK assays, whereas amprenavir and eletriptan both also have experimental (PAMPA-SDM) P_0_ values. The results for loperamide, in contrast, are considered less precise because of uncertainty in predicted P_0_ values. The P_0_ for loperamide could not be determined via MDCK or PAMPA assays and, as such, is based on a SDM prediction. Although SDM predictions based on experimental descriptors can be fairly accurate, experimental P_0_ are more reliable. The uncertainty in the P_0_ prediction for loperamide consequently leads to greater uncertainty in its fitted J_pgp,active,max_ values. Though experimental P_0_ values are generally reliable, they are still subject to error. The experimental P_0_ values for quinidine, amprenavir and eletriptan could still result in an estimation that is off by one order of magnitude. Thus, the maximal J_pgp,active_ determined from these fits are fairly consistent within the inextricable error that comes with P_0_ values.

This maximum J_pgp,active_ of 1.6 × 10^−4^ μmol/cm^2^/s obtained from the Michaelis–Menten fits for four borderline compounds represents the energy threshold for efflux transport determined for the MDCKII-MDR1 cells utilized in this work. Essentially: it is unlikely that highly membrane-permeable compounds with greater flux demands for its effective efflux would be affected by any efflux transporter. As previously mentioned, compounds that are located within the borderline window of membrane permeability may exhibit efflux, depending on their concentration.

### 4.6. Linking a Passive Membrane Permeability Threshold to the Energy Limit

While the J_pgp,active_ value is interesting, it must be linked with a more accessible metric such as membrane permeability to be useful in practice. As described earlier, for high ER J_pgp,active_ ≈ J_passive,in_, meaning almost all compound passively diffusing into the cell is actively transported out again. If the membrane poses the main resistance, J_passive,in_ corresponds to P_m_×C_ext_. However, this assumption is not valid for low ER values, or if other transport resistances are dominant. We thus used our model to calculate the maximum P_m_ that would lead to a specific ER assuming a maximal J_pgp,active_ of 1.6 × 10^−4^ μmol/cm^2^/s as described in Section 3.2.5, see also Supplementary Materials S1 (Section S16).

**Figure 7:**
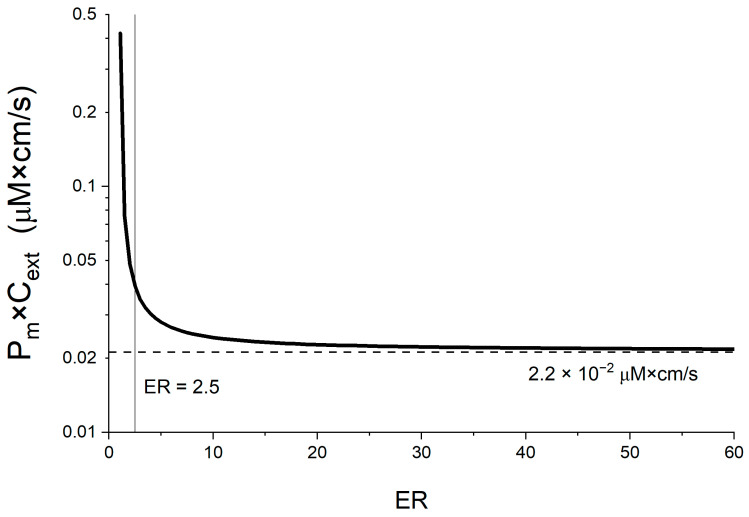
**The P_m_×C_ext_ threshold associated with the maximal J_pgp,active_ value of 1.6 × 10^−4^ μmol/cm^2^/s as a function of ER values.** The threshold lies at a value of log(P_m_×C_ext_) = −1.7.

As is evident from Figure 7, the P_m_×C_ext_ threshold stays constant across all ER values, except in the lower ranges. To appreciate why this is, it must be considered that at low ER values where P-gp activity is diminished, the passive backflow of the compound from the cytosol is significant. This does not apply for compounds exhibiting high ER values where P-gp is more active, see Supplementary Materials S1 (Section S17). However, this affects only a small fraction of the significant efflux ratios in our database, specifically those that just exceed the significance threshold. Therefore, the plateau remains the most interesting value. However, this sharp increase in the P_m_×C_ext_ limit for low ER values might explain the few remaining outliers that did indeed show low ER values (2.6–2.8). Especially when one considers that low recovery may also have resulted in an overestimation of ER.

### 4.7. Sensitivity Analysis of P_m_ Threshold

Although the energy threshold should remain static among different compounds and concentrations, the conversion of J_pgp,active_ into a P_m_ value is influenced by several factors. Firstly, the apical membrane surface area factor SA is expected to be quite consequential, as this directly affects the maximal J_pgp,active_ value (see Figures S11–S13). Fortunately, SA does not affect the extracted P_m_ threshold if we consider the maximal J_pgp,active_ that corresponds with the respective SA. For example, if the SA is increased by 3.2 (as is the case with 7.5 vs. 24), the extracted J_pgp,active_ is also increased by a factor of 3.2, but the extracted P_m_×C_ext_ threshold is not affected (see Figure S10). Regardless of which surface area is assumed, saturation always occurs for the same set of permeable compounds at the same concentration, so the limiting value of permeability × concentration remains constant. The empirical determination of the P_m_×C_ext_ threshold specific to our cells is thus not affected by the uncertainty of the SA factor. However, if a fixed maximal J_pgp,active_ is used to extract P_m_, the resulting values will scale directly with SA (see Figure S11), which is of relevance if the threshold is determined by theoretical deliberations on the energy limit, and not empirically. This becomes critical when the energy limit is determined independently (e.g., from ATP-turnover rates, see Supplementary Materials S1 (Section S2)) because SA variability would then introduce a major source of uncertainty.

Secondly, as stated in the preceding section, the maximal J_pgp,active_ presented in this work was determined specifically for MDCK-MDR1 cells. The maximum flux value can vary among different cell types. Since the energy needs of MDCK cells are higher than that of other, less active cells (see Supplementary Materials S1 (Section S2)), they likely have a higher energy limit than the average cell. Therefore, it is essential to note that variations will exist between different cell types. Cells with increased energy demands/production are likely to exhibit a higher maximum flux value. For example, in cancer cells an upregulation of mitochondrial respiration has been reported [87], which would also suggest an increased J_pgp,active,max_. The observation that inhibition of the electron transport chain restored the cells’ drug sensitivity [87] is fully consistent with our assumption of an energy limitation.

The energy limit of the cells, and thus their maximal J_pgp,active_, was found to be directly proportional to the corresponding P_m_ threshold. The observed variability in maximal J_pgp,active_ (range 8.7 × 10^−6^ to 4.4 × 10^−4^ μmol/cm^2^/s) thus directly translates to a range of the log(P_m_×C_ext_) threshold of −2.94 to −1.23. An increase in maximum flux shifts the logP_m_ threshold higher by the same factor. Therefore, it is important to reiterate that the P_m_ threshold provided here is not a universal figure, as it was established for the MDCK-MDR1 cells available to us. Consequently, the same P_m_ cut-off cannot be applied uniformly across all cell types. For instance, cells in the blood–brain barrier (BBB) exhibit greater activity compared to Caco-2 cells because they have high expression of various efflux transporters to tightly regulate brain access [88]. Thus, if BBB cells demonstrate a greater maximum flux value, it stands to reason that this would enable the effective efflux of compounds with increased passive membrane permeability. The higher density of mitochondria in the blood–brain barrier [89] may result in a higher energetic limit (and thus higher P_m_×C_ext_ threshold). This—together with the typically lower compound concentrations in blood compared to the intestine—may explain why certain compounds exhibit high bioavailability but low brain uptake, if they are not actively effluxed in the gut but are in the brain (even though passive membrane permeability is similar in the gut and the brain [90]).

Finally, the importance of concentration for the P_m_ threshold was very apparent. As expected it was found that the P_m_ threshold is indirectly proportional to the concentration, since their product determines the passive diffusive flux rate through the membrane that counteracts active transport. As the concentration of the compound decreases, the threshold rises (and the opposite is true for increasing concentrations). This is why the metric proposed for the threshold is not simply P_m_, but rather the P_m_-concentration product, P_m_×C_ext_ (µM cm/s).

The effect of paracellular transport, compound charge, as well as basolateral uptake on the P_m_×C_ext_ threshold was also evaluated, but was found to be inconsequential (Figures S12–S14). Thus, the final log(P_m_×C_ext_) threshold for our MDCK-MDR1 cell system— determined empirically with borderline compounds—was found to lie at −1.7. This is slightly higher than the threshold considered initially for the identification of outliers, and as such did not result in the re-identification of more outliers not previously considered.

### 4.8. Re-Evaluation of Literature Data

**Figure 8:**
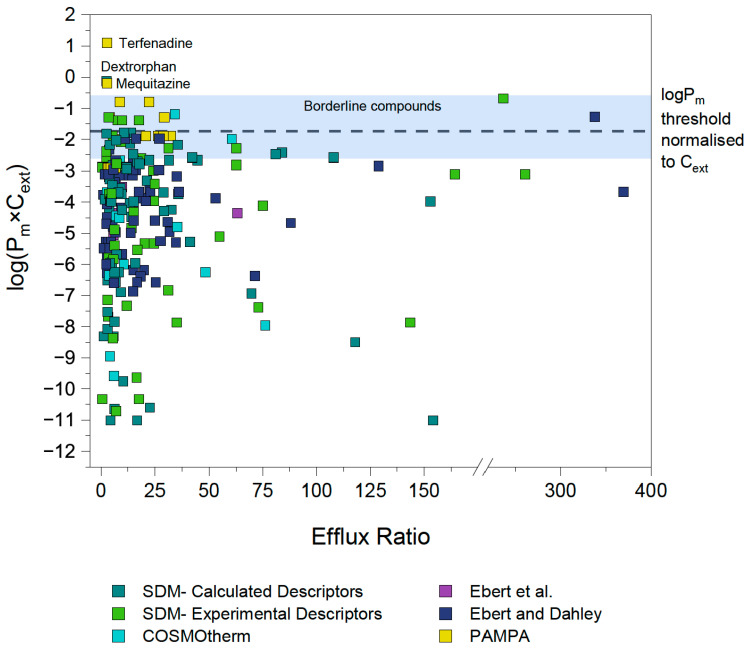
**log(P_m_×C_ext_) vs. ER of re-evaluated data from all transporter (P-gp, BCRP and MRP2) datasets.** The log(P_m_ ×C_ext_) threshold for the MDCKII-MDR1 cells was determined to be −1.7. With the exception of the three indicated outlier compounds, no compounds with P_m_ ×C_ext_ above 2.2 × 10^−2^ cm/s×μM showed significant efflux. The efflux of borderline compounds is highly dependent on concentration. Experimental P_m_ values sourced from Ebert et al. [32] and Ebert and Dahley [26].

294 significant ER values from MDCK assays were gathered from 46 different sources in the literature. Based on the spread of the starting experimental data, it was initially hypothesized that for MDCK cells, compounds with a P_m_×C_ext_ value exceeding 1 × 10^−2^ cm/s would be too membrane-permeable for the cell to maintain effective efflux against their high rates of passive diffusion. Two-thirds of the data did not contradict this hypothesis from the outset. In other words, these compounds were confirmed efflux substrates with a P_m_×C_ext_ lower than 1 × 10^−2^ cm/s. Figure 8 shows the distribution of the data of all three transporters after re-evaluation of the outliers, along with the final P_m_×C_ext_ threshold line of 2.2 × 10^−2^ cm/s (and borderline area) that was determined empirically in this study.

The depicted P_m_×C_ext_ threshold is based on the J_pgp,active,max_ of 1.6 × 10^−4^ μmol/cm^2^/s determined for our MDCK-MDR1 cells. Due to normalization of the threshold P_m_ to external concentration, it is independent of assay concentration used.

**Figure 9:**
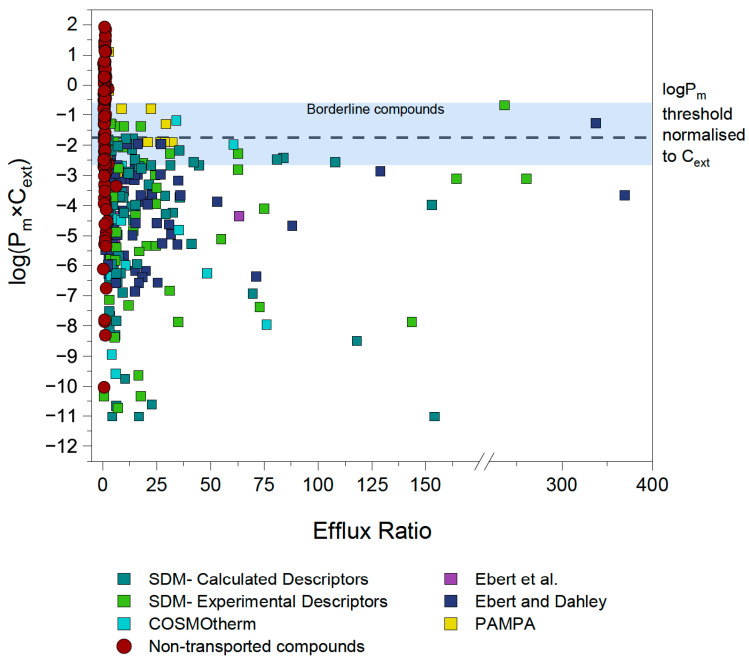
**log(P_m_×C_ext_) vs. ER of re-evaluated data from all transporter (P-gp, BCRP and MRP2) datasets, along with non-transported compounds in red.** Experimental P_m_ values sourced from Ebert et al. [32] and Ebert and Dahley [26].

It is remarkable that among all compounds analyzed here and documented in the literature as being substrates of one of the three primary efflux transporters associated with MDR, all have P_m_×C_ext_ values either below the threshold, or they fall in the borderline window and exhibit only concentration-dependent efflux. This supports the theory that there is indeed a P_m_ limit for active efflux—and due to it being based on the fundamental principle of cellular energy constraints, that it is universal for three different transporters. Ultimately, there are only three unconvincing, low ER compounds which have a P_m_×C_ext_ value exceeding the threshold and its corresponding borderline window. This is notable as there is no shortage of pharmaceutical compounds with log(P_m_×C_ext_) values above the threshold. As described in Section 3.2.1, 123 compounds with non-significant ER values were also evaluated. Figure 9 depicts the P_m_×C_ext_ values for these compounds with ER values less than 2.5 in red, and as is evident about 60% of them fall above the threshold. Compounds below the threshold may potentially be affected by efflux transport, and in vitro transwell assays for these compounds are warranted. However, compounds above the threshold are not expected to be affected by efflux transporters, and assays to check for an ER value would be unnecessary.

Our results align with the Biopharmaceutics Drug Disposition Classification System (BDDCS), which categorizes drugs into four classes according to solubility and intestinal permeability. According to Benet et al. [91], efflux transport should be considered for Class II compounds (low solubility, high passive permeability), whereas membrane transporters—both uptake and efflux—play a critical role for Class III (high solubility, low permeability) and Class IV (low solubility, low permeability) drugs. In contrast, for Class I drugs (high solubility, high permeability), active transport is generally not relevant. This is fully consistent with our defined P_m_×C_ext_ threshold, since we would not anticipate active transport under conditions of high permeability combined with high concentrations. 

## 5. Conclusions

In this study, we propose an energy limit for active efflux, which can be translated into a membrane permeability threshold to simplify identification of compounds that may exhibit significant efflux. We collected nearly 300 ER values from the MDCK assays of three transporters (P-gp, BCRP and MRP2) from the existing literature to investigate this premise. After establishing a preliminary threshold, a systematic analysis of outliers (compounds with ER values > 2.5 and log(P_m_×C_ext_) values > −2) led to the reclassification of most outliers as conformers. Concentration-dependent transwell assays for borderline compounds enabled the more precise identification of the maximal flux value (J_pgp,active,max_) of 1.6 × 10^−4^ μmol/cm^2^/s for our MDCK-MDR1 cell systems. It was found that the energy limit seems to be enforced by the cell through the mechanism of saturation. The maximal J_pgp,active_ was then translated into a more accessible threshold P_m_ value. The threshold P_m_ was normalized to external concentration for a concentration-independent P_m_×C_ext_ threshold. This P_m_×C_ext_ threshold falls within the mid-range of typical hydrophobicity/lipophilicity values for most chemicals of interest, making it relevant across a broad spectrum of compounds.

Our results confirm that passive membrane permeability (P_m_×C_ext_) can be used as a filtering metric to determine whether the pharmacokinetics of pharmaceutical compounds can be affected by efflux. However, accurate P_m_ values are critical for this determination. The model should be applicable to other cell lines or tissues, as the underlying principle—that active transport must counteract passive permeation and that energy consumption is inherently limited—is universal to all cells. However, the specific threshold will need to be determined for each cell type, as different cells have different energy budgets. One approach could be to estimate the threshold directly from the energy budget of the considered cell types, although parameters such as surface area, stoichiometry, and ATP turnover introduce additional uncertainties, and the practicability of this approach will need to be evaluated in future studies. A more direct and robust alternative is to determine the precise P_m_×C_ext_ threshold experimentally using borderline compounds on the cell line of interest, as we did in this study, which minimizes uncertainties related the above-mentioned factors.

The new approach would reduce the need for time- and resource-intensive transport assays for compounds above the threshold and would assist with the design of in vitro experiments that avoid saturation effects, thereby minimizing false negatives. In addition, it can aid rational lead optimization by allowing drugs of interest to be engineered for desired transporter interactions through adjustments in passive membrane permeability. A key practical limitation—shared with in vitro assays—is that the freely dissolved concentration (e.g., in the gut for oral absorption studies or in blood for blood–brain barrier studies) must be known, as it directly influences the threshold. We recognize that in physiological scenarios, multiple transporters may act synergistically, cellular energy may fluctuate under pathological conditions, and genetic polymorphisms can alter transporter activity. Our model assumes constant stoichiometry for ATP-dependent efflux transporters; under this assumption, synergistic transport by different transporter types does not change the total energy required to transport a given number of molecules. Nevertheless, variations in stoichiometry, energy limitation, or polymorphisms could alter the effective threshold, representing important limitations and avenues for future investigation. In the future, our quantitative framework may provide a basis for refining strategies to prevent active efflux through induced intracellular ATP depletion [92,93,94,95], although such applications remain to be explored. Overall, this novel mechanistic approach, grounded in cellular energy constraints, offers a promising way to predict when active efflux is unlikely solely from the chemical structure.

## Data Availability

The datasets used and/or analyzed during the current study are available from the corresponding author upon request.

## Supplementary Materials

The following supporting information can be downloaded at: https://www.mdpi.com/article/10.3390/pharmaceutics17111455/s1. Figure S1: Physiological and cellular approaches towards calculating theoretical maximal flux values; Figure S2: Apparent permeability and efflux ratios as a function of concentration for the compounds acebutolol, amprenavir, loperamide, eletriptan, nelfinavir, prazosin and quinidine; Figure S3: Three-compartment model simulations of the concentration over time in the different compartments in the B → A direction; Figure S4: P-gp facilitated flux fit values versus concentration for seven compounds with S = 1; Figure S5: P-gp facilitated flux fit values versus concentration for seven compounds with S = 7.5; Figure S6: P-gp facilitated flux fit values versus concentration for seven compounds with S = 24; Figure S7: Michaelis-Menten fits of J_pgp,active_ values for apical surface membrane factor = 1 and mean maximal J_pgp,active_ values; Figure S8: Michaelis-Menten fits of J_pgp,active_ values for apical surface membrane factor = 7.5 and mean maximal J_pgp,active_ values; Figure S9: Michaelis-Menten fits of J_pgp,active_ values for apical surface membrane factor = 24 and mean maximal J_pgp,active_ values; Figure S10: The P_m_×C_ext_ threshold as a function of ER values extracted from our experiments assuming different SA, as indicated; Figure S11: The P_m_×C_ext_ threshold associated with a fixed maximal J_pgp,active_ value of 1.6 × 10^−4^ μmol/cm^2^/s as a function of ER values for different SA; Figure S12: The P_m_×C_ext_ threshold associated with a fixed maximal J_pgp,active_ value of 1.6 × 10^−4^ μmol/cm^2^/s as a function of ER values based on magnitude of basolateral uptake transporter activity; Figure S13: The P_m_×C_ext_ threshold associated with a fixed maximal J_pgp,active_ value of 1.6 × 10^−4^ μmol/cm^2^/s as a function of ER values based on the whether the compound is charged or neutral; Figure S14: The P_m_×C_ext_ threshold associated with a fixed maximal J_pgp,active_ value of 1.6 × 10^−4^ μmol/cm^2^/s as a function of ER values based on the magnitude of paracellular transport; Table S1: Pgp dataset: Chemical properties and P_m_ values; Table S2 BCRP dataset: Chemical properties and calculated P_m_ values; Table S3 MRP2 dataset: Chemical properties and calculated P_m_ value; Table S4 Concentration-corrected P_m_ values for all transporters; Table S5 P_m_ values calculated from PAMPA K_hex/w_ values; Table S6 Outliers analysis; Table S7 Compounds with insignificant ER values: Chemical properties and P_m_ values; Table S8 Concentration-corrected P_m_ values for compounds with insignificant ER values. References [96,97,98,99,100,101,102,103,104,105,106,107,108,109,110,111,112,113,114,115] are only cited in the Supplementary Materials.
