# Supplementary material for: Classifying Effluxable Versus Non-Effluxable Compounds Using a Permeability Threshold Based on Fundamental Energy Constraints
Source: Pharmaceutics. 2025 Nov 11;17(11):1455. doi: 10.3390/pharmaceutics17111455 (PMC12655371; doi:10.3390/pharmaceutics17111455)
Supplement: Supplementary file 1 [file pharmaceutics-17-01455-s001.zip › supplementary materials/Supplementary Materials S1.pdf]

Supplementary Materials S1

for

Classifying Effluxable versus Non- Effluxable Compounds Using a Permeability Threshold Based on Fundamental Energy Constraints

Authors and Affiliations

Sone Kotze [a], Kai-Uwe Goss [a,b], Andrea Ebert [a,*],

[a] Department of Computational Biology and Chemistry, Helmholtz Centre for Environmental Research (UFZ), Permoserstraße 15, 04318 Leipzig, Germany
[b] Institute of Chemistry, University of Halle-Wittenberg, Kurt-Mothes-Straße 2, 06120 Halle, Germany
[*] corresponding author

Table of Contents

List of Figures 2

List of Equations 2

1. Model Equations 3
2. Theoretical Calculation of $J_{pgp,active}$ 6
3. Chemicals and reagents 8
4. Experimental conditions, recovery, apparent permeabilities and calculated P_0 and $logK_{hex/w}$ values from PAMPA experiments 10
5. Apparent permeabilities, recoveries and ER from MDCK-MDR1 bidirectional assays 11
6. Apparent permeabilities, recoveries and ER from MDCK-MDR1 bidirectional concentration dependence assays 12
7. Three-compartment model simulations 15
8. Reanalysis of concentration-dependent measurements from literature 16
9. Fitting of PS_{pgp} values fixed parameters 16
10. Fitting of PS_{pgp} values for apical surface membrane factor = 1 17
11. Fitting of PS_{pgp} vales for apical surface membrane factor = 7.5 19
12. Fitting of PS_{pgp} values for apical surface membrane factor = 24 21
13. Michaelis-Menten fits of $J_{pgp,active}$ values for apical surface membrane factor = 1 and mean maximal $J_{pgp,active}$ values 23
14. Michaelis-Menten fits of $J_{pgp,active}$ values for apical surface membrane factor = 7.5 and mean maximal $J_{pgp,active}$ values 24
15. Michaelis-Menten fits of $J_{pgp,active}$ values for apical surface membrane factor = 24 and mean maximal $J_{pgp,active}$ values 25

16. Linking maximal $J_{pgp,active}$ values to permeability 26
17. Rationalization of the raised threshold observed for low ER values 27
18. Sensitivity analysis 27

List of Figures

Figure S1: Physiological and cellular approaches towards calculating theoretical maximal flux values. .. 6

Figure S2: Apparent permeability and efflux ratios as a function of concentration for the compounds acebutolol, amprenavir, loperamide, eletriptan, nelfinavir, prazosin and quinidine. 14
Figure S3: Three-compartment model simulations of the concentration over time in the different compartments in the B → A direction. 15
Figure S4: P-gp facilitated flux fit values versus concentration for seven compounds with S =1. 17
Figure S5: P-gp facilitated flux fit values versus concentration for seven compounds with S = 7.5. 19
Figure S6: P-gp facilitated flux fit values versus concentration for seven compounds with S = 24. 21
Figure S7: Michaelis-Menten fits of $J_{pgp,active}$ values for apical surface membrane factor = 1 and mean maximal $J_{pgp,active}$ values. 23
Figure S8: Michaelis-Menten fits of $J_{pgp,active}$ values for apical surface membrane factor = 7.5 and mean maximal $J_{pgp,active}$ values. 24
Figure S9: Michaelis-Menten fits of $J_{pgp,active}$ values for apical surface membrane factor = 24 and mean maximal $J_{pgp,active}$ values. 25
Figure S10: The P_m*C_{ext} threshold as a function of ER values extracted from our experiments assuming different SA, as indicated.. 26
Figure S11: The P_m*C_{ext} threshold associated with a fixed maximal $J_{pgp,active}$ value of 1.6 x 10^{-4} µmol/cm^2/s as a function of ER values for different SA. 27
Figure S12: The P_m*C_{ext} threshold associated with a fixed maximal $J_{pgp,active}$ value of 1.6 x 10^{-4} µmol/cm^2/s as a function of ER values based on magnitude of basolateral uptake transporter activity.. 28 **Figure S13:** The P_m*C_{ext} threshold associated with a fixed maximal $J_{pgp,active}$ value of 1.6 x 10^{-4} µmol/cm^2/s as a function of ER values based on the whether the compound is charged or neutral. 28
Figure S14: The P_m*C_{ext} threshold associated with a fixed maximal $J_{pgp,active}$ value of 1.6 x 10^{-4} µmol/cm^2/s as a function of ER values based on the magnitude of paracellular transport. 29

List of Equations

S1 $P_{trans,A\rightarrow B}$ 4
S2 $P_{trans,B\rightarrow A}$ 4
S3 $P_{app,A\rightarrow B}$. 5
S4 $P_{app,B\rightarrow A}$. 5
S5 ER 5
S6 $C_{cyt,a}$ 5
S7 $C_{ABL,a}$ 5
S8 C_{filter} 5

1. Model Equations

Model equations for perfect sink conditions (C_b=0) are directly taken from Kotze et al. [21]. More details and the derivation can be looked up there. The following abbreviations were used in this section: **List of mathematical abbreviations**

Abbreviation	Description	Reference
C_a	Bulk concentration on the apical side	
$C_{ABL,a}$	ABL concentration on the apical side adjacent to the apical membrane	
$C_{ABL,b}$	ABL concentration on the basolateral side adjacent to the filter	
C_b	Bulk concentration on the basolateral side	
$C_{cyt,a}$	Cytosolic concentration adjacent to the apical membrane	
$C_{cyt,b}$	Cytosolic concentration adjacent to the basolateral membrane	
C_{filter}	Filter concentration adjacent to the basolateral membrane	
D_{cyt}	Diffusion coefficient through cytosol: $D_{cyt} = D_w \cdot 0.05$	Calculation according to Verkman [96]
D_w	Diffusion coefficient through water: $D_W = 1.348 \cdot 10^{(-4.13-0.453 \cdot \log MW)}$	Calculation according to Avdeef et al. [97]
ER	Efflux ratio: $P_{app,B\to A}/P_{app,A\to B}$	
$f_{n,cyt}$	Fraction of neutral species in the cytosol.	Calculation according to Dahley et al. [98]
$f_{n,a}$	Fraction of neutral species on the apical side	Calculated according to Henderson [99], Hasselbalch [100] and Escher et al. [24]
$f_{n,b}$	Fraction of neutral species on the basolateral side	See above
$P_{app,A\to B}$	Experimentally-obtained apparent permeability in the apical to basolateral direction	
$P_{app,B\to A}$	Experimentally obtained apparent permeability in the basolateral to apical direction	
$P_{ABL,a}$	Permeability of both species through the apical ABL: $P_{ABL,a} = D_w/x_{ABL,a}$	
$P_{ABL,b}$	Permeability of both species through the basolateral ABL: $P_{ABL,b} = D_w/d_{ABL,b}$	
P_{cyt}	Permeability of both species through the cytosol: $P_{cyt}=D_{cyt}/x_{cyt}$	Bittermann and Goss [29]

P_{filter}	Permeability of both species through the filter	Karlsson et al. [101]
$P_{m,a}$	Permeability of the neutral species through the apical membrane: $PS_{m,a} = P_0 * SA * f_n$	
$P_{m,b}$	Permeability of the neutral species through the basolateral membrane: $PS_{m,b} = P_0 * f_n$	
P_0	Intrinsic membrane permeability of the neutral species	
P_{para}	Paracellular transport, assumed equal in both directions	
$P_{pgp,app}^{active}$	Apparent permeability of P-gp-mediated efflux: $P_{pgp} * fraction\ of\ species\ Pgp\ acts\ on$	
SA	Surface Area factor of the apical membrane due to microvilli, assumed 1, 7.5 or 24	
$P_{ABL,b+filter}$	Permeability of filter and basolateral ABL combined, equal to $P_{ABL,b} * P_{filter}/(P_{ABL,b} + P_{filter})$	
$P_{b,app}^{active}$	Apparent permeability of basolateral transporter-mediated influx	
$P_{trans,A\rightarrow B}$	Apparent transcellular permeability, including active transport, diffusion across membranes, and diffusion across the cytosol, in A→B direction	
$P_{trans,B\rightarrow A}$	Apparent transcellular permeability, including active transport, diffusion across membranes, and diffusion across the cytosol, in B→A direction	

Model of transcellular permeability in both directions:

$$P_{trans,A\rightarrow B} = \frac{1}{\left(1 + \frac{P_{pgp,app}^{active}}{P_0 * SA * f_{n,cyt}}\right) * \left(\frac{1}{f_{n,a} * P_0} + \frac{1}{\frac{f_{n,a}}{f_{n,cyt}} * P_{cyt}}\right) + \frac{1}{P_0 * SA * f_{n,a}}} \quad \text{S1}$$

$$P_{trans,B\rightarrow A} = \left(1 + \frac{P_{pgp,app}^{active}}{P_0 * SA * f_{n,cyt}}\right) * \left(1 + \frac{P_{b,app}^{active}}{f_{n,b} * P_0}\right) * \frac{f_{n,b}}{f_{n,a}} * P_{trans,A\rightarrow B} \quad \text{S2}$$

Model of apparent permeability in both directions:

$$P_{\mathrm{app,A\rightarrow B}} = \frac{1}{\frac{1}{\frac{(P_{\mathrm{trans,A\rightarrow B}} + P_{\mathrm{para}})}{(P_{\mathrm{trans,B\rightarrow A}} + P_{\mathrm{para}})} * P_{\mathrm{ABL,b}}} + \frac{1}{\frac{(P_{\mathrm{trans,A\rightarrow B}} + P_{\mathrm{para}})}{(P_{\mathrm{trans,B\rightarrow A}} + P_{\mathrm{para}})} * P_{\mathrm{filter}}} + \frac{1}{(P_{\mathrm{trans,A\rightarrow B}} + P_{\mathrm{para}})} + \frac{1}{P_{\mathrm{ABL,a}}}}$$	S3

$$P_{\mathrm{app,B\rightarrow A}} = \frac{\frac{(P_{\mathrm{trans,B\rightarrow A}} + P_{\mathrm{para}})}{(P_{\mathrm{trans,A\rightarrow B}} + P_{\mathrm{para}})}}{\frac{1}{\frac{(P_{\mathrm{trans,A\rightarrow B}} + P_{\mathrm{para}})}{(P_{\mathrm{trans,B\rightarrow A}} + P_{\mathrm{para}})} * P_{\mathrm{ABL,b}}} + \frac{1}{\frac{(P_{\mathrm{trans,A\rightarrow B}} + P_{\mathrm{para}})}{(P_{\mathrm{trans,B\rightarrow A}} + P_{\mathrm{para}})} * P_{\mathrm{filter}}} + \frac{1}{(P_{\mathrm{trans,a\rightarrow b}} + P_{\mathrm{para}})} + \frac{1}{P_{\mathrm{ABL,a}}}}$$	S4

Modeled efflux ratio:

$$\mathrm{ER} = \frac{\frac{\left(1 + \frac{P_{\mathrm{pgp,app}}^{\mathrm{active}}}{f_{\mathrm{n,cyt}} * P_0 * 24}\right) * \left(1 + \frac{P_{\mathrm{b,app}}^{\mathrm{active}}}{f_{\mathrm{n,b}} * P_0}\right) * \frac{f_{\mathrm{n,b}}}{f_{\mathrm{n,a}}}}{\left(1 + \frac{P_{\mathrm{pgp,app}}^{\mathrm{active}}}{P_0 * \mathrm{SA} * f_{\mathrm{n,cyt}}}\right) * \left(\frac{1}{f_{\mathrm{n,a}} * P_0} + \frac{1}{\frac{f_{\mathrm{n,a}}}{f_{\mathrm{n,cyt}}} * P_{\mathrm{cyt}}}\right) + \frac{1}{P_0 * \mathrm{SA} * f_{\mathrm{n,a}}}} + P_{\mathrm{para}}}{\frac{1}{\left(1 + \frac{P_{\mathrm{pgp,app}}^{\mathrm{active}}}{P_0 * \mathrm{SA} * f_{\mathrm{n,cyt}}}\right) * \left(\frac{1}{f_{\mathrm{n,a}} * P_0} + \frac{1}{\frac{f_{\mathrm{n,a}}}{f_{\mathrm{n,cyt}}} * P_{\mathrm{cyt}}}\right) + \frac{1}{P_0 * \mathrm{SA} * f_{\mathrm{n,a}}}} + P_{\mathrm{para}}}$$	S5

Modeled local compound concentrations:

$$C_{\mathrm{cyt,a}} = \frac{P_0 * SA * f_{n,a} * C_{\mathrm{ABL,a}} + \frac{P_0 * f_{n,b} * P_{cyt} + P_{\mathrm{b,app}}^{\mathrm{active}} * P_{cyt}}{f_{n,cyt} * P_0 + P_{cyt}} * C_{\mathrm{filter}}}{f_{n,cyt} * P_0 * SA + P_{\mathrm{pgp,app}}^{\mathrm{active}} + P_{cyt} * \frac{f_{n,c} * P_0}{f_{n,c} * P_0 + P_{cyt}}}$$	S6

$$C_{\mathrm{ABL,a}} = \frac{P_{ABL,a} * (P_{trans,B\rightarrow A} + P_{para} + P_{ABL,b+filter}) * c_a}{P_{ABL,a} * (P_{trans,B\rightarrow A} + P_{para} + P_{ABL,b+filter}) + P_{trans,A\rightarrow B} * (P_{ABL,b+filter}) + P_{\mathrm{para}} * P_{ABL,b+filter}}$$	S7

$$C_{\mathrm{filter}} = \frac{(P_{trans,a\rightarrow b} + P_{para})}{P_{trans,b\rightarrow a} + P_{para} + P_{ABL,b+filter}} * C_{\mathrm{ABL,a}}$$	S8

2. Theoretical Calculation of $J_{pgp,active}$

Starting out with a simplified theoretical calculation enables, at the very least, the ascertainment of the general range or starting estimate for the energy limit. As such, a rather crude calculation was performed by obtaining a value for the energy production of the cell — expressed through ATP turnover, assuming 1:1 stoichiometry of drug transport and ATP hydrolysis (this assumption may be an underestimation, since reports indicate that 1–3 ATP molecules may be consumed per transported substrate [102]). As such the theoretical $J_{pgp,active,max}$ might be overestimated by up to a factor of 3, however, this does not affect the experimental determination of $J_{pgp,active,max}$ presented in the main work. Figure S1 shows how this can be approached through measurements of oxygen or glucose consumption on a physiological or cellular level. Three different sources of ATP turnover were used [103-105]. On a physiological level, the production was pegged by Flamholz et al. [105] at a value of 5 x 10^{-5} ATP molecules per cell, which translates to about 8.3 x 10^{-17} mol/s (moles of ATP per second). Using the same principles, values from Dunn and Grider [103] generated a similar rate of 2 x 10^{-17} mol/s. Both of these estimates were originally enumerated for the entire human body, and then calculated per cell by assuming that there are about ~ 10^{13} cells in the body (this figure excludes red blood cells, which contain no mitochondria and make up ~ 70% of all cells) [105, 106].

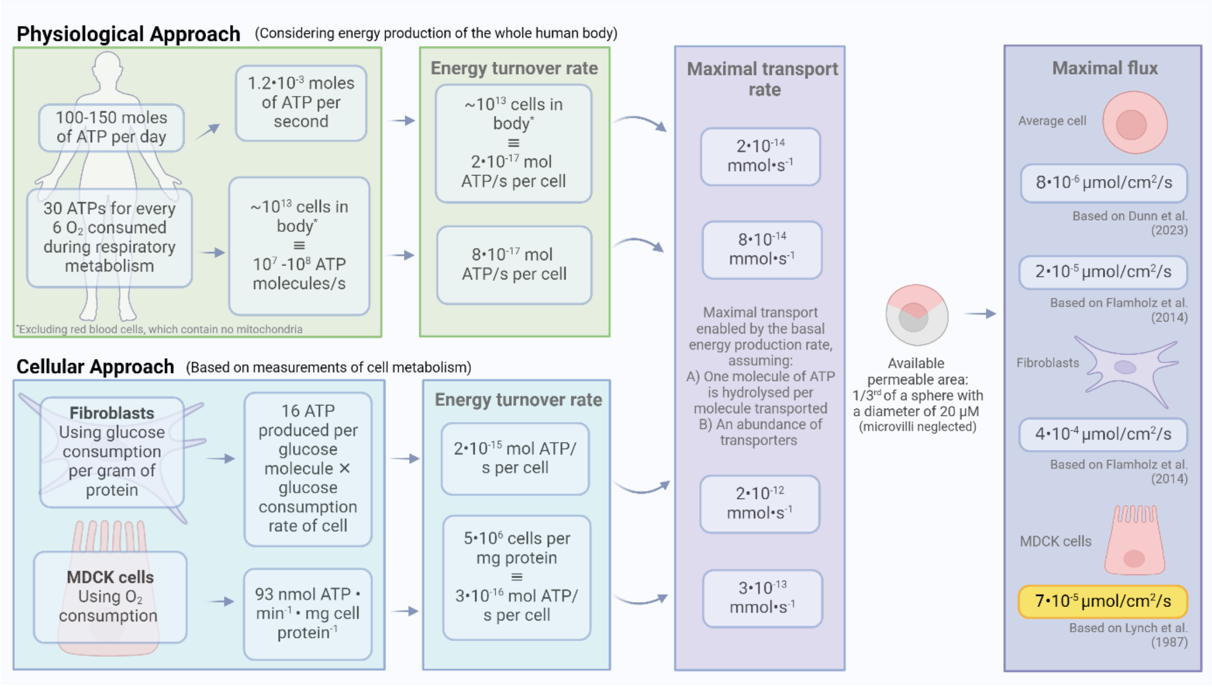

Figure S1:Physiological and cellular approaches towards calculating theoretical maximal flux values. The maximal efflux that can be facilitated by cells is estimated from the rate of ATP production per cell and the surface area of a cell available for permeation. Since different cells have different energy demands depending on their location and function, the maximal flux value will differ between cells.

As such, these estimated ATP production rates are an average value for all cells. However, it is worth remembering that different cells may have varying energy consumption based on their location and function. For example, muscle and brain cells have much higher energy needs (and thus more mitochondria) than adipocytes or skin cells. ATP production values were found for fibroblasts [15], which have a very high energy demand when they are in the activated state. Indeed, they were estimated to

have a higher maximal transport rate than the general cell averages above, calculated at 2 x 10^{-15} mol/s. Since this study deals with MDCKII cells, an ATP production rate determined specifically for these cells [14] was used, and it was also about factor 10 higher than the averages reported above at 3 x 10^{-16} mol/s.

This is not surprising, since it could be expected that MDCK cells require more energy than the average cell due to their increased metabolic and active transport rates, and the fact that they also need to maintain their polarised state. If it is assumed that one molecule of ATP is hydrolysed for every one molecule transported [107] (and assuming sufficient amounts of transporter proteins in the cell), then an ATP production rate of 3 x 10^{-16} mol/s would enable a maximal transport rate of 3.1 x10^{-13} mmol/s. The surface area of the cell was then estimated as one third of the surface area of a sphere with a diameter of 20 µm (neglecting the microvilli). This results in an estimate for the maximal flux in MDCK cells of 7.4 x 10^{-5} µmol/cm^2/s. The preceding calculation is highly simplified, and the simplifications will result in errors at each step which might skew the values. Any error in the ATP turnover rate or stoichiometry will propagate linearly into the results. However, such a theoretical estimation is merely an attempt to get an order-of-magnitude estimate as a comparison for experimental values.

3. Chemicals and reagents

Chemical	Supplier
Cell culture	
Dulbecco's modified Eagle medium (DMEM) (1X) + GlutaMAX™-I	Life Technologies Ltd., Paisley, UK
Foetal bovine serum (FBS)	Life Technologies Corporation, New York, USA
100 U/mL penicillin and 100 µg/mL streptomycin	Life Technologies Corporation, New York, USA
MDCK Bidirectional Transport Assays	
Hank's balanced salt solution (HBSS)	Biowest SAS, Nuaillé, France
Lucifer Yellow CH dilithium salt	Sigma-Aldrich, Co., St. Louis, MO, USA
N-(2-Hydroxyethyl)piperazine-N'-(2-ethanesulfonic acid) (HEPES)	Sigma-Aldrich, Co., St. Louis, MO, USA
Acebutolol hydrochloride	Sigma-Aldrich, Co., St. Louis, MO, USA
Amprenavir	Sigma-Aldrich, Co., St. Louis, MO, USA
Brompheniramine maleate	HPC Standards GmBH, Bordsdorf, Germany
Chlorpheniramine maleate	HPC Standards GmBH, Bordsdorf, Germany
Chlorpromazine hydrochloride	Sigma-Aldrich, Co., St. Louis, MO, USA
Clemastine fumarate	Sigma-Aldrich, Co., St. Louis, MO, USA
Clomipramine hydrochloride	Merck KGaA, Darmstadt, Germany
Desipramine hydrochloride	Sigma-Aldrich, Co., St. Louis, MO, USA
Diphenhydramine chloride	Sigma-Aldrich, Co., St. Louis, MO, USA
Doxylamine succinate	Sigma-Aldrich, Co., St. Louis, MO, USA
Eletriptan hydrobromide	Sigma-Aldrich, Co., St. Louis, MO, USA
Emetine dihydrochloride	Sigma-Aldrich, Co., St. Louis, MO, USA
Fluoxetine hydrochloride	Dr. Ehrenstorfer GmbH, LGC Limited, Teddington, UK
Loratadine	Sigma-Aldrich, Co., St. Louis, MO, USA
Loperamide hydrochloride	Sigma-Aldrich, Co., St. Louis, MO, USA
Nelfinavir	AdipoGen Life Sciences, Inc., San Diego, CA, USA
Prazosin hydrochloride	Thermo Fisher Scientific, Waltham, MA, USA
Pyrilamine maleate	Sigma-Aldrich, Co., St. Louis, MO, USA
Quinidine	Sigma-Aldrich, Co., St. Louis, MO, USA
Reserpine	Sigma-Aldrich, Co., St. Louis, MO, USA
Ritonavir	Sigma-Aldrich, Co., St. Louis, MO, USA
Sertraline hydrochloride	Sigma-Aldrich, Co., St. Louis, MO, USA
Terfenadine	Sigma-Aldrich, Co., St. Louis, MO, USA
PAMPA	
β-Alanine	Sigma-Aldrich, Co., St. Louis, MO, USA
4-Morpholineethanesulfonic acid (MES)	Sigma-Aldrich, Co., St. Louis, MO, USA
N-[Tris(hydroxymethyl)methyl]-3-aminopropanesulfonic acid (TAPS)	Sigma-Aldrich, Co., St. Louis, MO, USA
3-(N-Morpholino)propanesulfonic acid (MOPS)	Sigma-Aldrich, Co., St. Louis, MO, USA

3-(Cyclohexylamino)-2-hydroxy-1-propanesulfonic acid (CAPSO)	Sigma-Aldrich, Co., St. Louis, MO, USA
Dimethyl sulfoxide (DMSO)	Th. Geyer GmbH & Co. KG, Renningen, Germany
Hexadecane	Acros Organics, Thermo Fisher Scientific, Waltham, MA, USA
LiChrosolv EtOH	Merck KGaA, Darmstadt, Germany
Amprenavir	Sigma-Aldrich, Co., St. Louis, MO, USA
Darifenacin hydrobromide	Sigma-Aldrich, Co., St. Louis, MO, USA
Desloratadine	Sigma-Aldrich, Co., St. Louis, MO, USA
Docetaxel hydrate	Cayman Chemicals, Ann Arbor, MI, USA
Emetine dihydrochloride	Sigma-Aldrich, Co., St. Louis, MO, USA
Erlotinib hydrochloride	ChemPUR GmbH, Karslruhe, Germany
Eletriptan hydrobromide	Sigma-Aldrich, Co., St. Louis, MO, USA
Gefitinib	Sigma-Aldrich, Co., St. Louis, MO, USA
Mequitazine	Sigma-Aldrich, Co., St. Louis, MO, USA
Nelfinavir	AdipoGen Life Sciences, Inc., San Diego, CA, USA
Prazosin hydrochloride	Thermo Fisher Scientific, Waltham, MA, USA
Phenelzine sulfate	British Pharmacopoeia Commission Laboratory, Teddington, London, UK
Terfenadine	Sigma-Aldrich, Co., St. Louis, MO, USA
Verapamil hydrochloride	Sigma-Aldrich, Co., St. Louis, MO, USA

4. Experimental conditions, recovery, apparent permeabilities and calculated P_0 and $logK_{hex/w}$ values from PAMPA experiments

Compound	Conc. [µg/mL]	Time [h]	pH	Replicate	Recovery [%]	Corrected $logP_{app}$	$logP_0$	$logK_{hex/w}$
Amprenavir	30	24	6.0	A	113	-6.23	-6.23	-1.75
				B	104	-6.16	-6.15	-1.68
				C	112	-6.23	-6.23	-1.75
Darifenacin	100	4	6.0	A	99	-5.71	-3.04	1.41
				B	100	-5.74	-3.06	1.38
				C	100	-5.74	-3.07	1.38
Desloratadine	200	4	6.0	A	89	-6.37	-2.44	1.94
				B	92	-6.33	-2.41	1.98
				C	92	-6.32	-2.40	1.98
Eletriptan	200	24	7.5	A	116	-6.07	-4.35	0.07
				B	114	-6.05	-4.34	0.09
				C	113	-6.06	-4.34	0.08
Emetine	600	2	7.0	A	107	-5.69	-3.34	1.13
				B	111	-5.73	-3.39	1.08
				C	109	-5.72	-3.38	1.09
Erlotinib	100	24	4.5	A	82	-5.86	-4.88	-0.46
				B	82	-5.93	-4.95	-0.52
				C	82	-5.94	-4.96	-0.54
Mequitazine	40	24	4.5	A	109	-6.49	-0.90	3.49
				B	107	-6.52	-0.92	3.46
				C	113	-6.52	-0.93	3.46
Nelfinavir	30	4	7.0	A	57	-4.99	-4.85	-0.35
				B	61	-5.09	-4.97	-0.47
				C	55	-5.02	-4.89	-0.39
Prazosin[a]	90	24	7.5	A	16	-7.85	-7.84	-3.41
				B	13	-7.92	-7.91	-3.48
				C	19	-7.95	-7.93	-3.51
Terfenadine	100	4	4.0	A	102	-5.59	0.04	4.51
				B	101	-5.58	0.06	4.52
				C	98	-5.53	0.11	4.57
Verapamil	500	4	5.0	A	91	-5.40	-1.69	2.76
				B	92	-5.41	-1.70	2.75
				C	92	-5.41	-1.70	2.76

[a] Extremely low recovery due to strong sorption effects, therefore not included in further evaluation. When ethanol extraction was also used in the donor compartment for extraction (not routinely done) recovery increased to 65 – 81 %

5. Apparent permeabilities, recoveries and ER from MDCK-MDR1 bidirectional assays

**Compound**	**Conc.** [µM]	$P_{app,A\rightarrow B}$ [10^{-6} cm/s]	**log** $P_{app,A\rightarrow B}$	**Recovery A ⟶ B** [%]	$P_{app,B\rightarrow A}$ [10^{-6} cm/s]	**log** $P_{app,B\rightarrow A}$	**Recovery B ⟶ A** [%]	**ER**
Amprenavir	20	5 ± 0.6	-5.3	96	138 ± 10	-3.9	88	28
Brompheniramine	6	134 ± 8	-3.9	63	121 ± 51	-3.9	83	0.9
Chlorpheniramine	7	141 ± 7	-3.9	65	118 ± 50	-3.9	83	0.8
Chlorpromazine	13	131 ± 16	-3.9	32	78 ± 44	-4.1	60	0.6
Clemastine	3	35 ± 4	-4.5	64	52 ± 31	-4.3	69	1.5
Clomipramine[a]	16	128 ± 17	-3.9	55	60 ± 29	-4.2	85	0.4
Desipramine	8	141 ± 14	-3.9	62	108 ± 45	-4.0	85	0.8
Diphenhydramine	8	171 ± 10	-3.8	83	295 ± 65	-3.5	79	1.7
Doxylamine	7	133 ± 13	-3.9	100	141 ± 39	-3.9	95	1.1
Emetine	10	10 ± 1	-5.0	52	104 ± 19	-4.0	82	11
Fluoxetine	6	35 ± 4	-4.5	68	49 ± 35	-4.3	60	1.4
Loratadine	10	127 ± 10	-3.9	58	86 ± 56	-4.1	77	0.7
Prazosin	5	44 ± 3	-4.4	81	138 ± 19	-3.9	83	3.2
Pyrilamine	7	182 ± 7	-3.7	100	187 ± 53	-3.7	101	1.0
Reserpine	7	24 ± 7	-4.6	97	62 ± 30	-4.2	90	2.6
Ritonavir	7	1 ± 0.2	-5.9	74	158 ± 77	-3.8	68	115
Sertraline	16	40 ± 12	-4.4	55	15 ± 11	-4.8	70	0.4
Terfenadine	11	12 ± 3	-4.9	79	33 ± 21	-4.5	60	2.8

[a] One replicate only

6. Apparent permeabilities, recoveries and ER from MDCK-MDR1 bidirectional concentration dependence assays

Compound	Conc.[a] [µM]	$P_{app,A\rightarrow B}$ [10^{-6} cm/s]	log $P_{app,A\rightarrow B}$	Recovery A ⟶ B [%]	$P_{app,B\rightarrow A}$ [10^{-6} cm/s]	log $P_{app,B\rightarrow A}$	Recovery B ⟶ A [%]	ER
Acebutolol	6	0.34	-6.47	88-95	7.20	-5.14	93-95	21
	12	0.32	-6.50	93-95	6.52	-5.19	92-96	21
	20	0.27	-6.57	88-92	8.40	-5.08	88-89	32
	60	0.31	-6.51	90-92	7.78	-5.11	92-93	25
	150	0.31	-6.51	91-93	8.37	-5.08	92-93	27
Amprenavir	0.2	16.7	-4.78	77-91	395	-3.40	106-109	24
	2	6.82	-5.17	103-112	184	-3.74	112-114	27
	10	6.54	-5.18	106-110	178	-3.75	110-116	28
	15	6.52	-5.19	106-112	157	-3.80	71-105	24
	15	8.95	-5.05	87-95	168	-3.77	92-105	19
	20	10.6	-4.98	78-89	197	-3.70	81-83	19
	30	11.0	-4.96	103-114	84.6	-4.07	85-95	8
	40	13.2	-4.88	92-98	92.6	-4.03	87-95	7
	50	15.6	-4.81	94-109	51.0	-4.29	82-90	3
	60	30.1	-4.52	94-95	30.0	-4.52	85-88	1
Eletriptan	0.1	9.70	-5.01	82-85	186	-3.73	69-71	19
	1	9.28	-5.03	87-91	200	-3.70	77-78	21
	10	13.6	-4.87	97-104	170	-3.77	84-86	13
	20	20.7	-4.68	99-102	115	-3.94	81-82	6
	40	28.6	-4.54	91	70.7	-4.15	78-79	3
Loperamide	0.1	10.3	-4.99	80-114	135	-3.87	113-127	13
	0.4	9.47	-5.02	81-89	122	-3.91	105-124	13
	4	24.1	-4.62	60-63	142	-3.85	88-91	6
	10	32.0	-4.49	69-113	100	-4.00	83-86	3
	20	65.2	-4.19	91-98	68.8	-4.16	87-91	1
Nelfinavir	5	6.87	-5.16	73-75	215	-3.67	81-99	31
	10	14.6	-4.84	87-91	373	-3.43	85-97	24
	20	16.1	-4.79	79-116	227	-3.64	84-86	14
	50	13.6	-4.87	64-69	53.8	-4.27	74-103	4
	100	10.4	-4.98	62-69	31.0	-4.51	82-91	3
Prazosin	0.03	65.0	-4.19	83-88	135	-3.87	86-87	2
	0.3	51.4	-4.29	82-84	154	-3.81	92-95	3
	3	63.2	-4.20	114-116	219	-3.66	100-105	4
	20	45.8	-4.34	86-87	98.0	-4.01	88-89	2
	40	34.7	-4.46	72-74	59.8	-4.22	84	2
Quinidine	0.05	19.8	-4.70	96-97	267	-3.57	85	14
	0.5	14.7	-4.83	105-108	280	-3.55	107-111	19
	1	22.5	-4.65	100-106	256	-3.59	104-109	11
	2	45.4	-4.34	94-95	229	-3.64	98-103	5
	5	41.2	-4.38	99-104	249	-3.60	112-116	6
	10	90.4	-4.04	98-103	163	-3.79	91-98	2
	15	128	-3.89	83-91	151	-3.82	95-97	1
	20	100	-4.00	117-119	148	-3.82	113-115	2
	50	136	-3.87	117-118	119	-3.92	114-117	1

[a] Refers to external concentration as applied in the assay

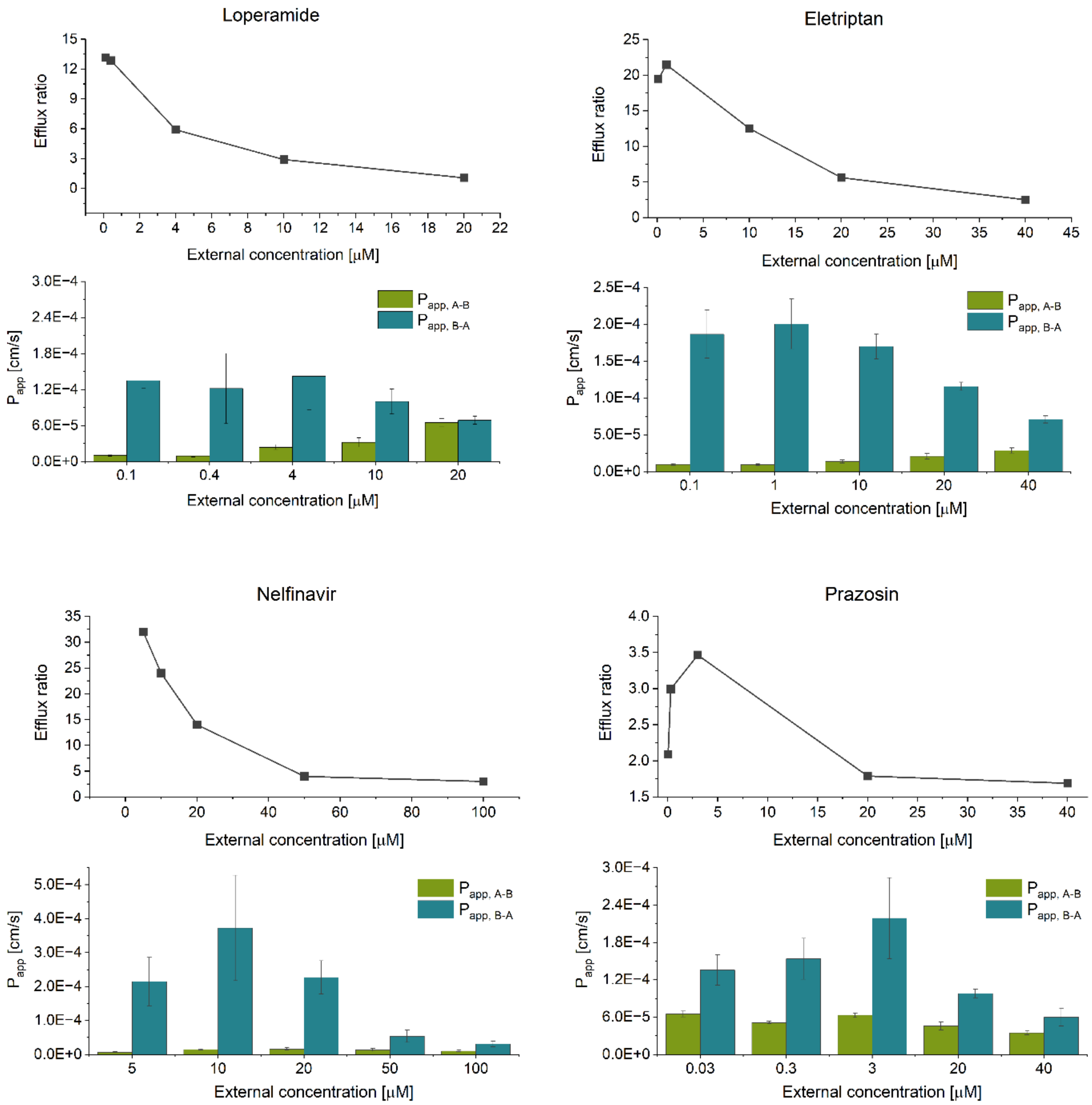

Loperamide
Efflux ratio
External concentration [µM]
P_app [cm/s]
P_app, A-B
P_app, B-A
External concentration [µM]
Eletriptan
Efflux ratio
External concentration [µM]
P_app [cm/s]
P_app, A-B
P_app, B-A
External concentration [µM]
Nelfinavir
Efflux ratio
External concentration [µM]
P_app [cm/s]
P_app, A-B
P_app, B-A
External concentration [µM]
Prazosin
Efflux ratio
External concentration [µM]
P_app [cm/s]
P_app, A-B
P_app, B-A
External concentration [µM]

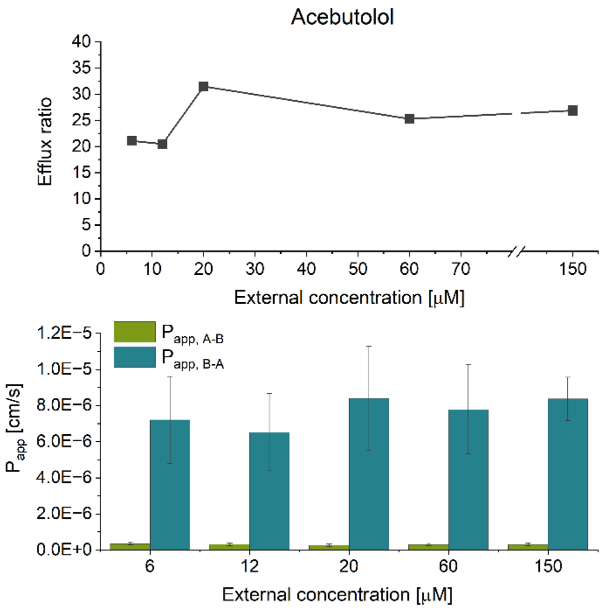

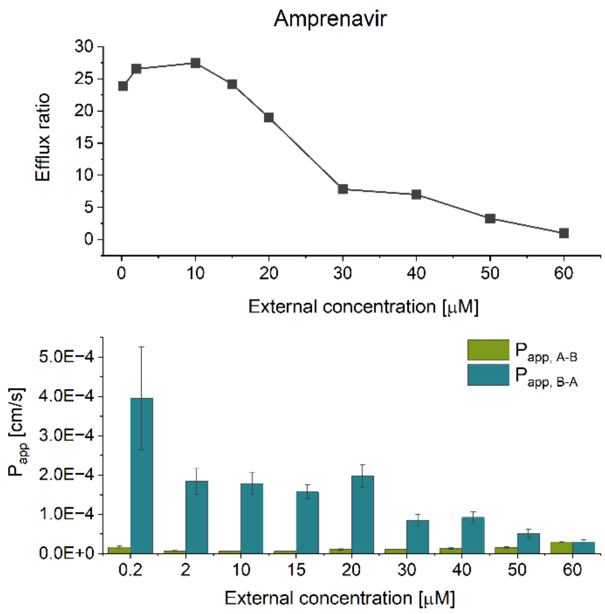

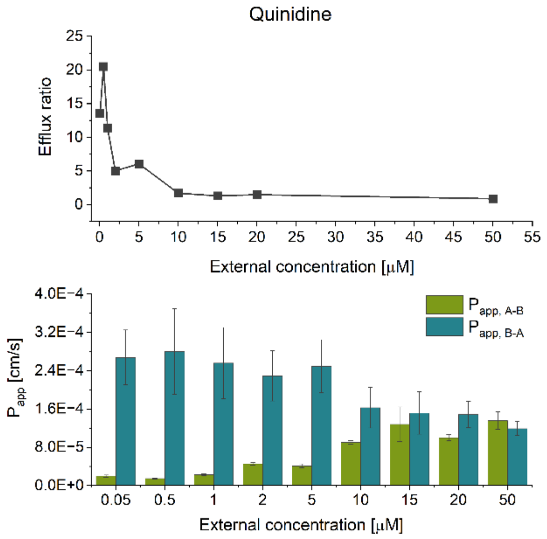

Figure S2: Apparent permeability and efflux ratios as a function of concentration for the compounds acebutolol, amprenavir, loperamide, eletriptan, nelfinavir, prazosin and quinidine. Error bars represent standard deviation. The ER (line graph, top panels) is the quotient of mean $P_{app,B \rightarrow A}$ over mean $P_{app,A \rightarrow B}$ values.

7. Three-compartment model simulations

While attempting to perform the SOLVER fits for the experimental P_{app} values, it was observed that the fits do not work as they should for the B⟶A direction if the apparent permeability for this direction is particularly fast. The $P_{app,\,B\longrightarrow A}$ values that were experimentally obtained for these compounds were often faster than what the inevitable limitation by the basolateral ABL would allow for. In these cases, the fits failed. To locate the root of this issue, the P_0 and lipid-water partition coefficient ($K_{lip/w}$) values for one of the compounds exhibiting this problem were used in a detailed three-compartment model [108], where the concentrations over time could be observed in each compartment. Figure S3 shows the concentration in the cytosol as a function of time in the B⟶A direction. From Figure S3 it can be seen that the cytosolic concentration rises and then drops again at each sampling timestep (every 480 seconds in this case).

A

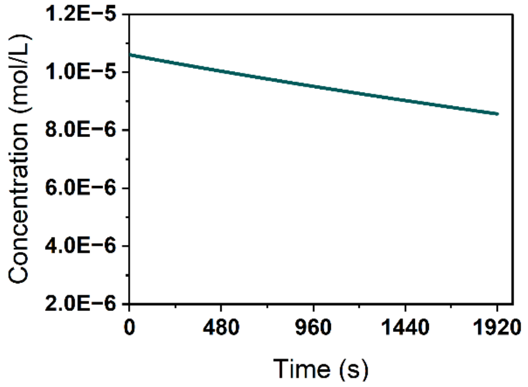

B

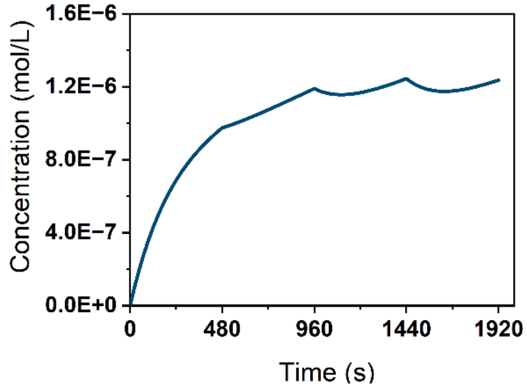

C

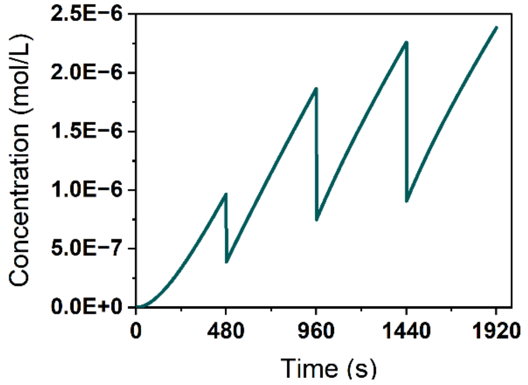

Figure S3: Three-compartment model simulations of the concentration over time in the different compartments in the B → A direction. A) Donor compartment, B) Cytosol C) Receiver compartment.

It was deduced that each time volume is sampled from the receiver compartment and replaced with fresh buffer, then the cytosol starts feeding the receiver compartment, which is why the cytosolic concentration drops at this very point. This is also evident when comparing the calculated P_{app} values with the experimentally-obtained ones. Initially the experimental P_{app} is slower than what we would expect mathematically, since the net rate into the cytosol is much higher than out of it, due to its sorption capacity. Then at some point the experimental P_{app} is faster than expected since the cytosol is no longer in steady-state with the other compartments, and the rate from the cytosol to the receiver compartment is higher than the rate from the donor to the cytosol. Ultimately, the observed trend in experimental data is actually predicted by the simulation. However, even though the problem is qualitatively obvious, a solution to avoid this issue experimentally could not be found. Increasing the time between sampling

steps would be one way of attempting to address it, but this would compromise the sink conditions. Furthermore, it is also not possible to merely take the first timesteps, as steady-state has not yet been reached in the initial stages.

These conclusions were drawn with the aid of the three-compartment model for the compound quinidine, however for the more permeable compounds used in this section, any point where the $P_{app,\, B\longrightarrow A}$ is faster than the basolateral ABL would technically allow for, it can be presumed that this is an artefact that is caused by this same issue. Ultimately what this means is that the B⟶A direction is simply as fast as it can possibly be, considering ABL-limitation. However, since these concentration-dependent experiments were also performed for one less permeable compound not near the threshold (acebutolol) it was possible to evaluate how the resultant flux values change when the fit works as expected. Acebutolol did not have the same issue that the other compounds have, with its measured $P_{app,\, B\longrightarrow A}$ never being faster than the calculated value. As such, the fit works in both directions, without any adjustments. The $J_{pgp,active}$ values for P-gp were then fitted both with and without fitting the B⟶A direction and compared, and it was observed that fitting the B⟶A direction does not make a significant difference to the end-result. As such, the final solution to circumventing the cytosol feeding issue was to only fit the A⟶B direction (assuming zero transport in the basolateral membrane) and evaluate P-gp flux only. In the absence of experimental workarounds, this was ultimately found to be the best solution. Nevertheless, the influence of basolateral transport cannot be completely ruled out, and some uncertainty remains. However, this uncertainty would persist even if the fit were applied in both directions, since, due to different local concentrations, saturation effects could occur in one direction but not in the other.

8. Reanalysis of concentration-dependent measurements from literature

As stated in the main text, Tachibana et al. [85] previously analyzed concentration-dependent measurements published by Shirasaka et al. [86] for a certain set of compounds with their own model. Unfortunately, reanalyzing the Shirasaka et al. data with our model did not yield reliable results as the evaluable concentration window was rather narrow. At higher concentrations, all compounds appeared to be limited by ABL effects (their data suggested a total ABL thickness of ~4 mm, compared to 426 µm in our experiments). As we were unfamiliar with their experimental setup, estimating paracellular transport was difficult. However, based on the predictions of Avdeef [97], paracellular transport could have affected P_{app} at low concentrations for both vinblastine and quinidine. Furthermore, while we could extract a total membrane thickness, the distribution of the ABL between the apical and basolateral sides is critical for the evaluation, and this information was unavailable. In addition, recovery data were missing. Collectively, these factors prevented us from reliably applying our model to their dataset.

9. Fitting of PS_{pgp} values fixed parameters

**$d_{ABL,apical}$ [cm]**	0.0133
**$d_{ABL,basolateral}$ [cm]**	0.0293
**d_{filter} [cm]**	1.15E-03
**$d_{cytosol}$ [cm]**	0.0015
**P_{para} factor**	0.1
**$pH_{e,apical}$**	7.4
**pH_i[a]**	7.69
**$pH_{e,basolateral}$**	7.4

[a]Calculated according to Dahley et al. [6]

10. Fitting of PS_{pgp} values for apical surface membrane factor = 1

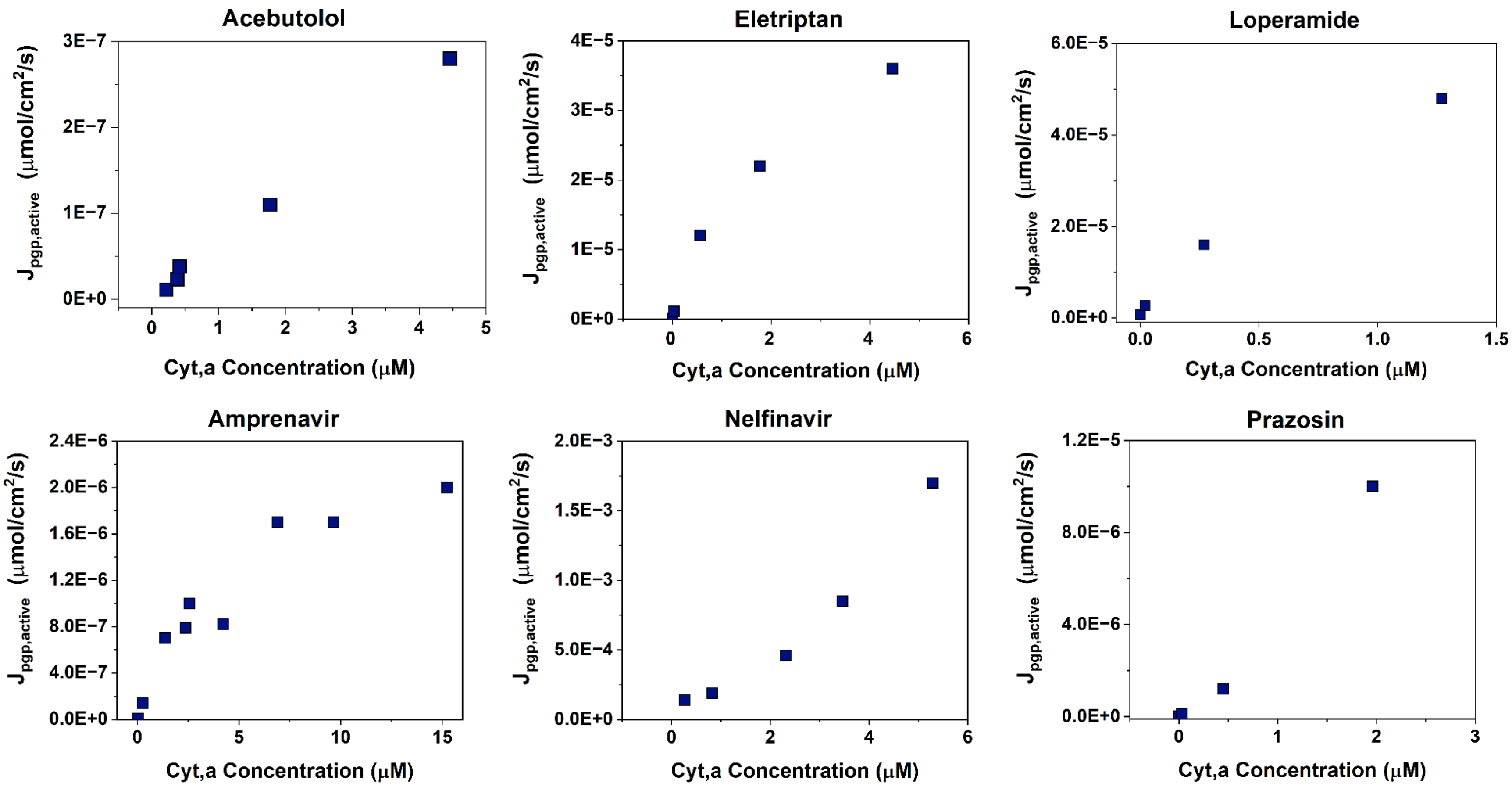

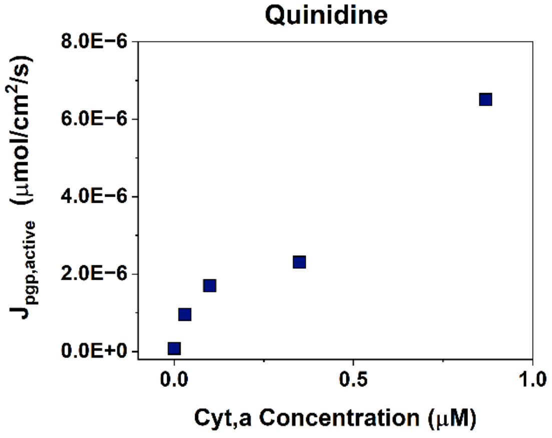

Figure S4: P-gp facilitated flux fit values versus concentration for seven compounds with S =1. Fits were performed for $J_{pgp,active}$ in the A ⟶ B direction with an apical surface membrane factor of 1, indicating no difference in surface area between the basolateral and apical membrane.

Compound	$P_{0,MDCK}$ [cm/s]	External concentration [µM]	Cyt,a concentration [µM]	PS_{pgp} [µmol/cm2/s]
Quinidine	1.71E-03	0.05	0.00	8.2E-08
		0.5	0.03	9.6E-07
		1	0.10	1.7E-06
		2	0.35	2.3E-06
		5	0.87	6.5E-06
Amprenavir	4.14E-05	0.2	0.05	6.0E-09
		2	0.28	1.4E-07
		10	1.38	7.0E-07
		15	2.56	1.0E-06
		15	2.37	7.9E-07
		20	4.23	8.2E-07
		30	6.91	1.7E-06
		40	9.65	1.7E-06
		50	15.24	2.0E-06
Eletriptan	4.57E-02	0.1	0.00	1.1E-07
		1	0.03	1.1E-06
		10	0.57	1.2E-05
		20	1.77	2.2E-05
		40	4.46	3.6E-05
Loperamide	8.61E-02	0.1	0.00	6.9E-07
		0.4	0.02	2.7E-06
		4	0.27	1.6E-05
		10	1.27	4.8E-05
Nelfinavir	1.56E-02	5	0.27	1.4E-04
		10	0.83	1.9E-04
		20	2.32	4.6E-04
		50	3.46	8.5E-04
		100	5.29	1.7E-03
Prazosin	1.71E-03	0.01	0.00	8.5E-09
		0.1	0.03	1.1E-07
		1	0.45	1.2E-06
		8	1.96	1.0E-05
Acebutolol	7.10E-05	6	0.22	1.1E-08
		12	0.38	2.3E-08
		20	0.42	3.8E-08
		60	1.76	1.1E-07
		150	4.46	2.8E-07

Factor of 2 applied to P_0

11. Fitting of PS_{pgp} vales for apical surface membrane factor = 7.5

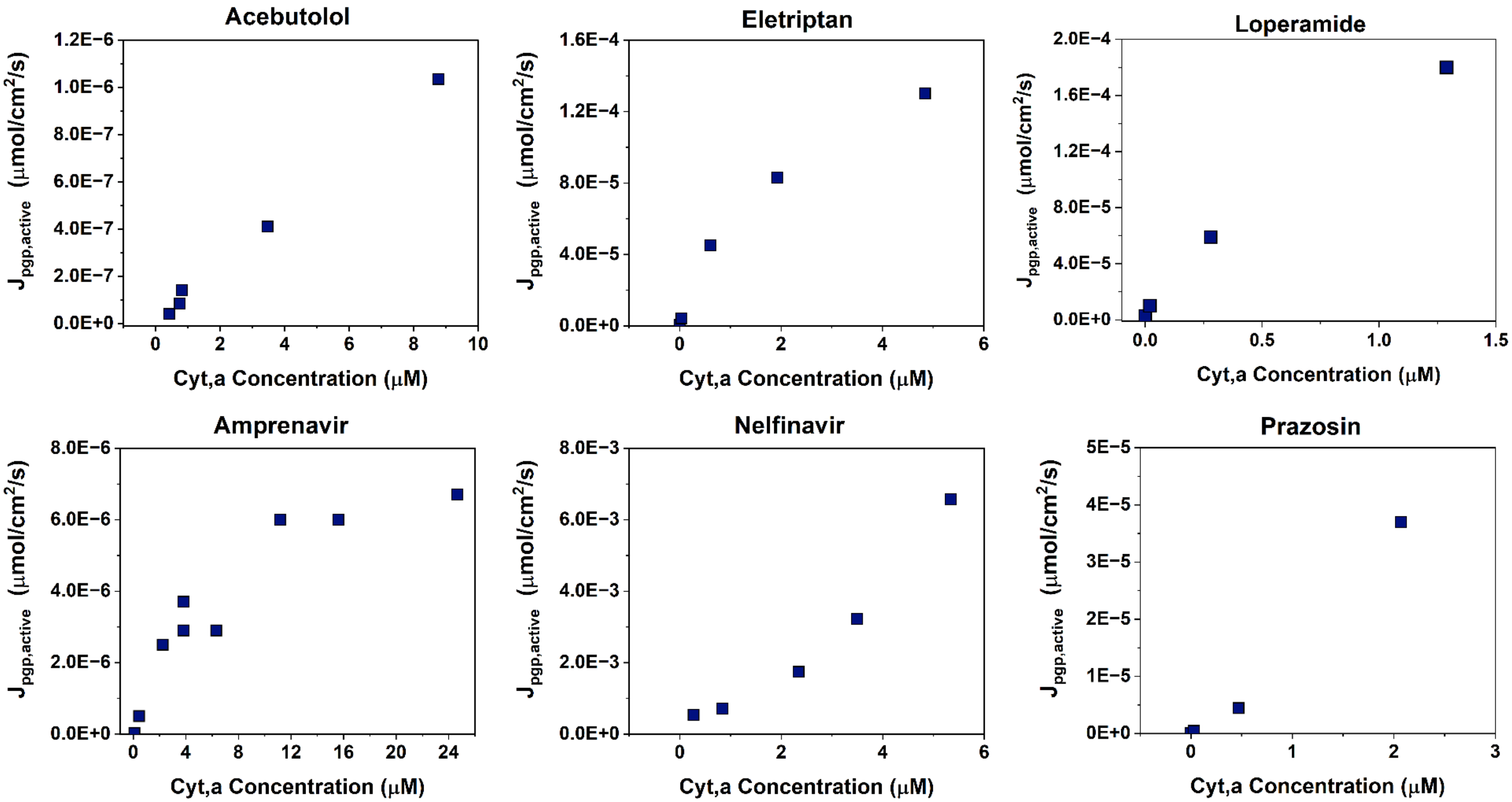

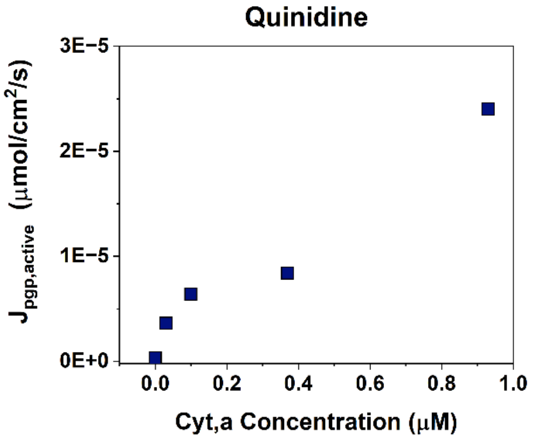

Figure S5: P-gp facilitated flux fit values versus concentration for seven compounds with S = 7.5. Fits were performed for $J_{pgp,active}$ in the A ⟶ B direction with an apical surface membrane factor of 7.5 as determined by Butor and Davoust [28].

**Compound**	$P_{0,MDCK}$ [cm/s]	**External concentration** [µM]	**Cyt,a concentration** [µM]	PS_{pgp} [µmol/cm2/s]
Quinidine	1.71E-03	0.05	0.00	3.1E-07
		0.5	0.03	3.6E-06
		1	0.10	6.4E-06
		2	0.37	8.4E-06
		5	0.93	2.4E-05
Amprenavir	4.14E-05	0.2	0.09	2.0E-08
		2	0.45	5.0E-07
		10	2.24	2.5E-06
		15	3.82	3.7E-06
		15	3.82	2.9E-06
		20	6.32	2.9E-06
		30	11.18	6.0E-06
		40	15.61	6.0E-06
		50	24.65	6.7E-06
Eletriptan	4.57E-02	0.1	0.00	4.0E-07
		1	0.04	4.2E-06
		10	0.61	4.5E-05
		20	1.93	8.3E-05
		40	4.84	1.3E-04
Loperamide	8.61E-02	0.1	0.00	2.6E-06
		0.4	0.02	1.0E-05
		4	0.28	5.9E-05
		10	1.29	1.8E-04
Nelfinavir	1.56E-02	5	0.27	5.3E-04
		10	0.84	7.1E-04
		20	2.34	1.7E-03
		50	3.49	3.2E-03
		100	5.34	6.6E-03
Prazosin	1.71E-03	0.01	0.00	3.1E-08
		0.1	0.03	4.1E-07
		1	0.47	4.4E-06
		8	2.07	3.7E-05
Acebutolol	7.10E-05	6	0.43	4.0E-08
		12	0.75	8.4E-08
		20	0.82	1.4E-07
		60	3.47	4.1E-07
		150	8.77	1.0E-06

12. Fitting of PS_{pgp} values for apical surface membrane factor = 24

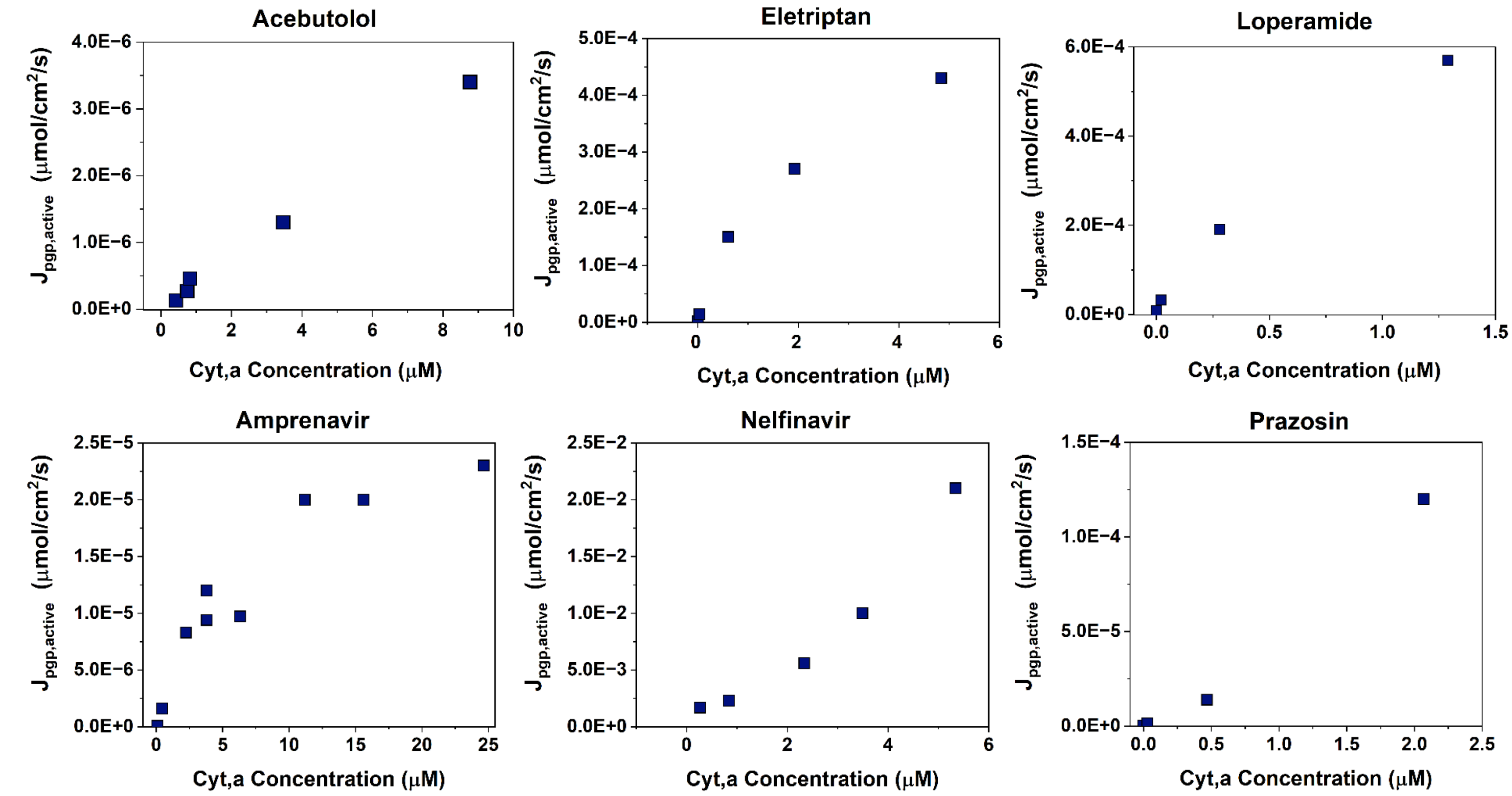

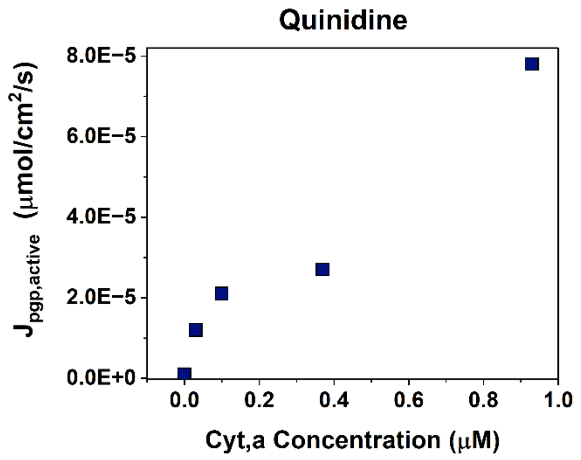

Figure S6: P-gp facilitated flux fit values versus concentration for seven compounds with S = 24. Fits were performed for $J_{pgp,active}$ in the A ⟶ B direction with an apical surface membrane factor of 24 as determined by Palay [27].

**Compound**	**$P_{0,MDCK}$** [cm/s]	**External concentration** [µM]	**Cyt,a concentration** [µM]	**PS_{pgp}** [µmol/cm2/s]
Quinidine	1.71E-03	0.05	0.00	9.9E-07
		0.5	0.03	1.2E-05
		1	0.10	2.1E-05
		2	0.37	2.7E-05
		5	0.93	7.8E-05
Amprenavir	4.14E-05	0.2	0.09	6.9E-08
		2	0.45	1.6E-06
		10	2.24	8.3E-06
		15	3.82	1.2E-05
		15	3.82	9.4E-06
		20	6.32	9.7E-06
		30	11.18	2.0E-05
		40	15.61	2.0E-05
		50	24.65	2.3E-05
Eletriptan	4.57E-02	0.1	0.00	1.3E-06
		1	0.04	1.4E-05
		10	0.61	1.5E-04
		20	1.93	2.7E-04
		40	4.84	4.3E-04
Loperamide	8.61E-02	0.1	0.00	8.3E-06
		0.4	0.02	3.2E-05
		4	0.28	1.9E-04
		10	1.29	5.7E-04
Nelfinavir	1.56E-02	5	0.27	1.7E-03
		10	0.84	2.3E-03
		20	2.34	5.6E-03
		50	3.49	1.0E-02
		100	5.34	2.1E-02
Prazosin	1.71E-03	0.01	0.00	1.0E-07
		0.1	0.03	1.3E-06
		1	0.47	1.4E-05
		8	2.07	1.2E-04
Acebutolol	7.10E-05	6	0.43	1.3E-07
		12	0.75	2.7E-07
		20	0.82	4.6E-07
		60	3.47	1.3E-06
		150	8.77	3.4E-06

13. Michaelis-Menten fits of $J_{pgp,active}$ values for apical surface membrane factor = 1 and mean maximal $J_{pgp,active}$ values

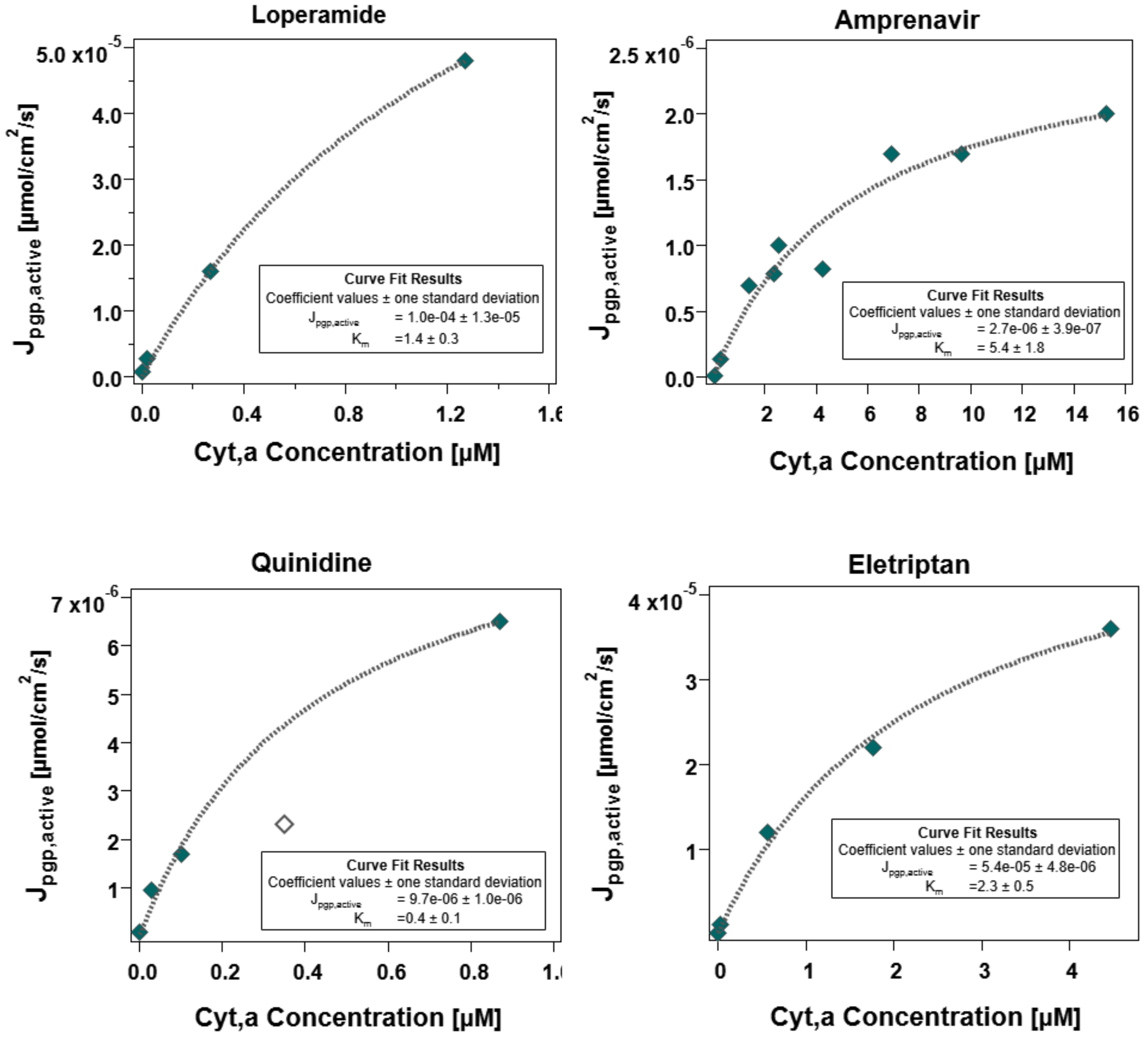

Figure S7: Michaelis-Menten fits of $J_{pgp,active}$ values for apical surface membrane factor = 1 and mean maximal $J_{pgp,active}$ values. For quinidine, the unfilled marker was not used in the fit.

Compound	$J_{pgp,active}$ [µmol/cm^2/s]	K_m
Loperamide	1.0E-04	0.3
Amprenavir	2.7E-06	5.4
Quinidine	9.7E-06	0.4
Eletriptan	5.4E-05	2.3
**Mean $J_{pgp,active}$**	**4.2E-05**	

14. Michaelis-Menten fits of $J_{pgp,active}$ values for apical surface membrane factor = 7.5 and mean maximal $J_{pgp,active}$ values

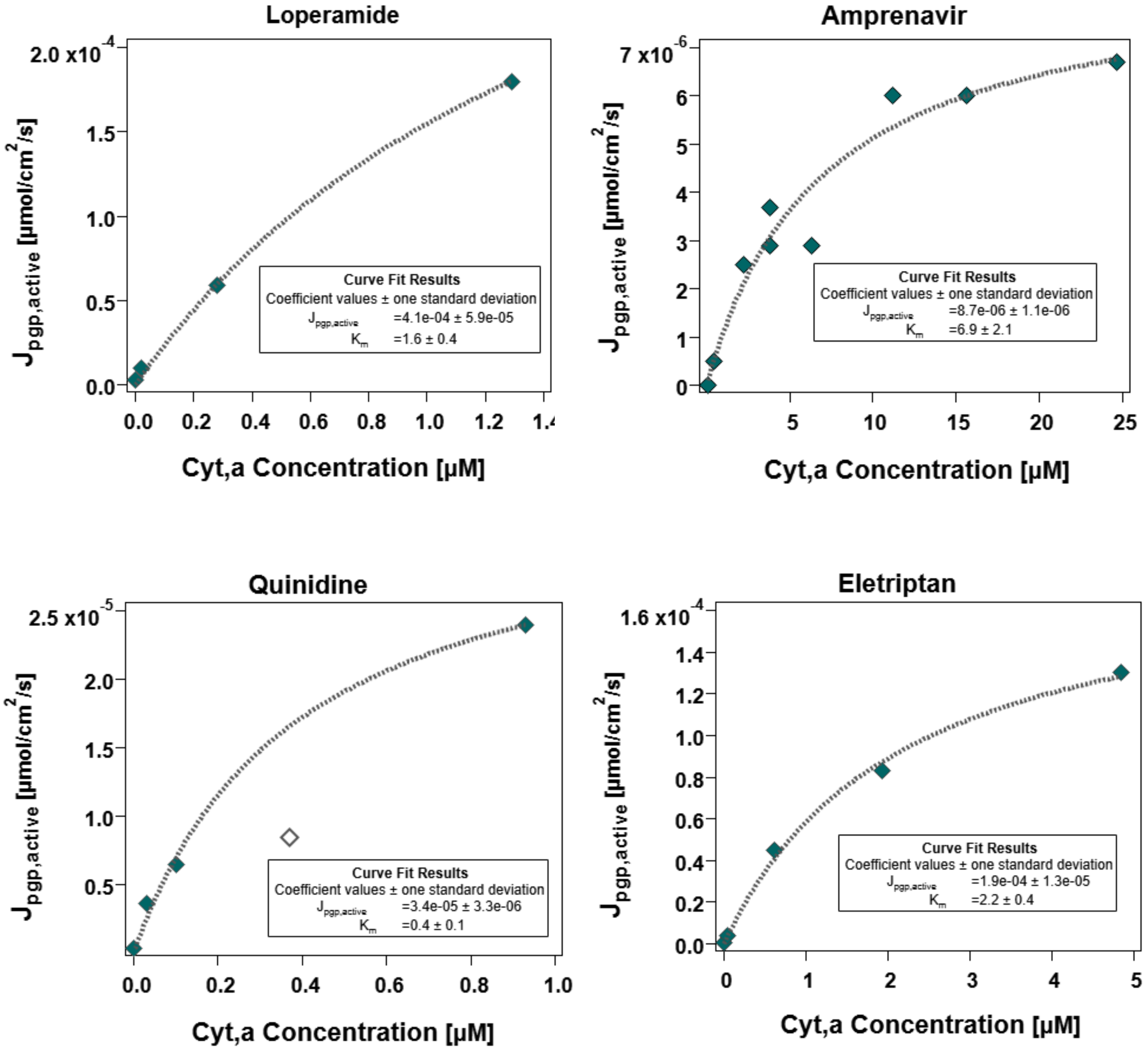

Figure S8: Michaelis-Menten fits of $J_{pgp,active}$ values for apical surface membrane factor = 7.5 and mean maximal $J_{pgp,active}$ values. For quinidine, the unfilled marker was not used in the fit.

**Compound**	$J_{pgp,active}$ [µmol/cm^2/s]	K_m
Loperamide	4.1E-04	1.6
Amprenavir	8.7E-06	6.9
Quinidine	3.4E-05	0.4
Eletriptan	1.9E-04	2.2
**Mean $J_{pgp,active}$**	**1.6E-04**	

15. Michaelis-Menten fits of $J_{pgp,active}$ values for apical surface membrane factor = 24 and mean maximal $J_{pgp,active}$ values

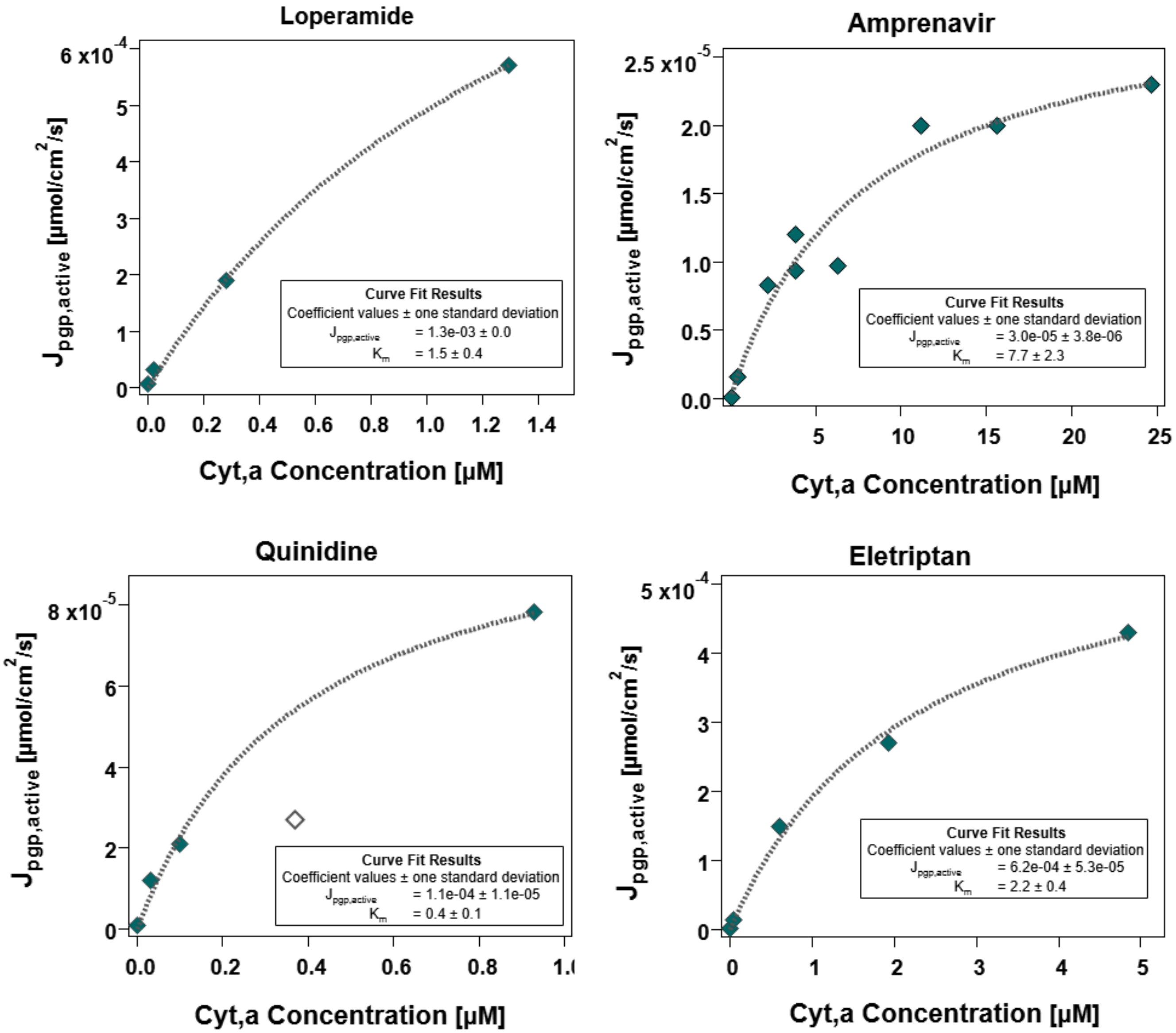

Figure S9: Michaelis-Menten fits of $J_{pgp,active}$ values for apical surface membrane factor = 24 and mean maximal $J_{pgp,active}$ values. For quinidine, the unfilled marker was not used in the fit.

Compound	$J_{pgp,active}$ [μmol/cm^2/s]	K_m
Loperamide	1.3E-03	1.5
Amprenavir	3.0E-05	7.7
Quinidine	1.1E-04	0.4
Eletriptan	6.2E-04	2.2
**Mean $J_{pgp,active}$**	**5.2E-04**	

16. Linking maximal $J_{pgp,active}$ values to permeability

To link maximal $J_{pgp,active}$ with P_m, the compound Quinidine (pKb1 = 8.31 and pKb2 = 4.19, molecular weight 324.417 g/mol) was used as a typical model compound in simulations. Passive diffusion through the aqueous layers is calculated from the thickness of the respective layer (x) and the diffusion coefficient of the compound in water ($\mathrm{D_w}$) which can be estimated from the molecular weight of the compound according to the relationship determined by Avdeef et al. [97, 109]. The permeability through the ABL, for example, is simply the quotient of these two factors, D_w/x . However, for other aqueous layers such as the filter, the reduced surface area available for permeation through the filter pores is also factored into P_{filter} [101]. For P_{cyt} it is instead the diffusion coefficient of the chemical in the cytosol ($\mathrm{D_{cyt}}$) that is used, which is estimated as one quarter of D_w [96]. Figure S10 depicts the concentration-corrected P_m (P_m*C_{ext}) value that was found to be associated with the respective determined maximal $J_{pgp,active}$ under the assumption that there is no difference between the apical and basolateral membrane surface areas, a difference of 7.5, or a difference of 24 respectively. While the extracted maximal $J_{pgp,active}$ strongly depend on SA, P_m*C_{ext} values for SA = 7.5 and SA = 24 overlap. Both are twice as large as the values for SA = 1. This factor of two arises because, for SA = 1, the experimental and calculated permeabilities had to be adjusted. The original experimental evaluation and the empirical correlation for SDM predictions [20] assumed the apical membrane resistance to be negligible compared to the basolateral resistance when assessing passive permeability. If, instead, two membranes must be traversed rather than one, the extracted P_0 for a single membrane must be multiplied by two.

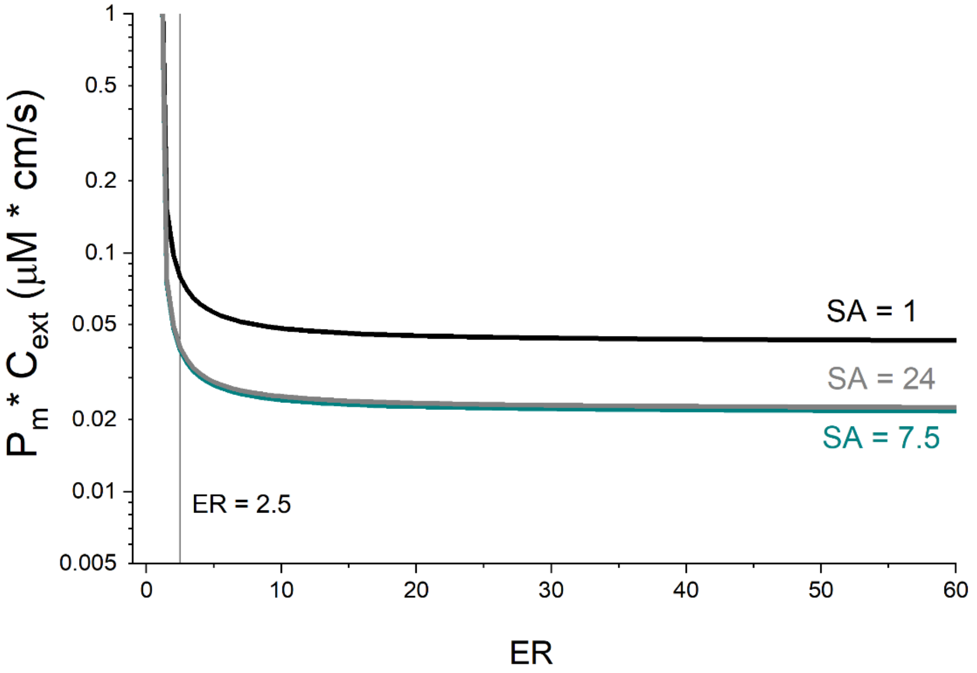

Figure S10: The P_m*C_{ext} threshold as a function of ER values extracted from our experiments assuming different SA, as indicated. Analysis was done using the respective $J_{pgp,active}$ values.

17. Rationalization of the raised threshold observed for low ER values

In the low ER value range, in order to double the ER, active efflux has to increase substantially to compete with the passive backflow. However, even with a substantial increase in P-gp activity, there is only a modest drop in cytosol concentration. Thus, in the low ER ranges, P0 has to decrease in order for the system to continue adhering to the maximal energy consumption value when the ER increases. In contrast, at higher ER values, active efflux dominates, making the passive backflow negligible. Doubling ER in this range involves proportionally smaller increases in P_{pgp} since it already vastly exceeds P_0, and active transport largely dictates the efflux. As a result, the cytosolic concentration decreases proportionally with the increase in P_{pgp} activity. Energy consumption remains the same despite the higher ER and reaches a plateau- leading to a concurrent Pm plateau. This is of course a simplified scenario, since the activity of the basolateral transporter and other factors also play a role—but it allows for a fundamental understanding of the phenomenon that can be observed in the lower ER ranges.

18. Sensitivity analysis

Figure S11 depicts the concentration-corrected P_m (P_m*C_{ext}) value that was found to be associated with the determined maximal $J_{pgp,active}$ assuming the different factor differences between the apical and basolateral membrane surface areas using a fixed maximal $J_{pgp,active}$ of 1.6 x 10^{-4} µmol/cm^2/s.

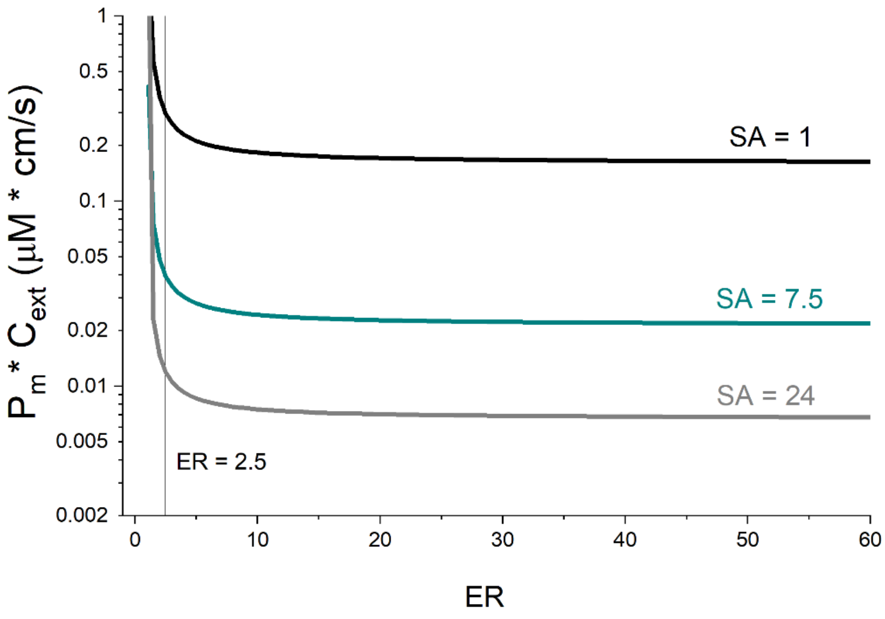

Figure S11: The P_m*C_{ext} threshold associated with a fixed maximal $J_{pgp,active}$ value of 1.6 x 10^{-4} µmol/cm^2/s as a function of ER values for different SA.

Figure S12 depicts the concentration-corrected P_m (P_m*C_{ext}) value that was found to be associated with the determined maximal $J_{pgp,active}$ of 1.6 x 10^{-4} µmol/cm^2/s (SA = 7.5) based on magnitude of basolateral uptake transporter activity.

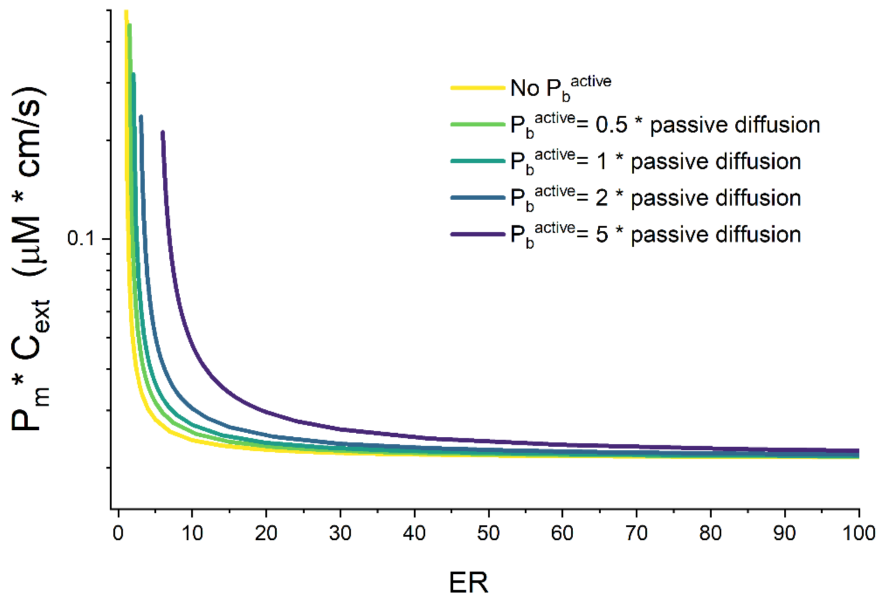

Figure S12: The P_m*C_{ext} threshold associated with a fixed maximal $J_{pgp,active}$ value of 1.6 x 10^{-4} µmol/cm^2/s as a function of ER values based on magnitude of basolateral uptake transporter activity.

Figure S13 depicts the concentration-corrected P_m (P_m*C_{ext}) value that was found to be associated with the determined maximal $J_{pgp,active}$ of 1.6 x 10^{-4} µmol/cm^2/s (SA = 7.5) based on whether the compound is charged or neutral.

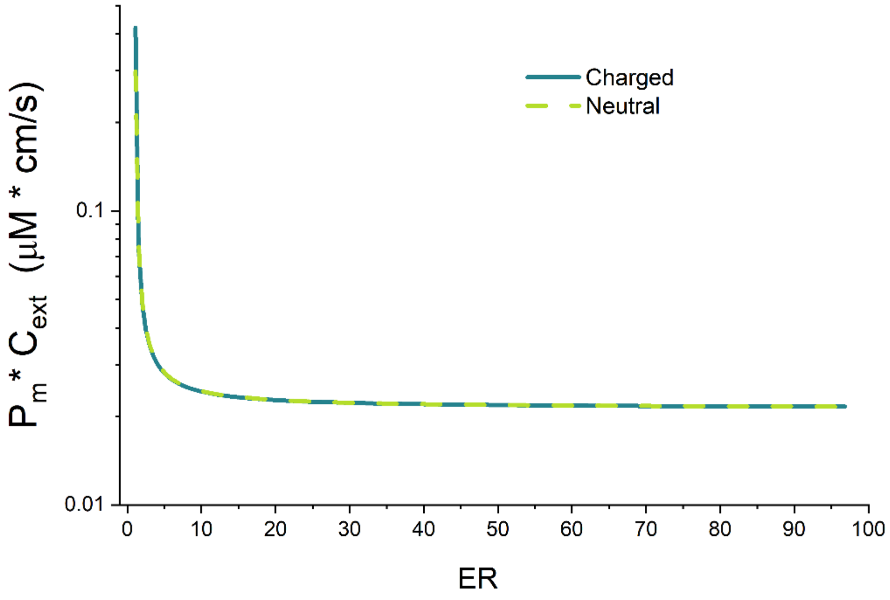

Figure S13: The P_m*C_{ext} threshold associated with a fixed maximal $J_{pgp,active}$ value of 1.6 x 10^{4} µmol/cm^2/s as a function of ER values based on the whether the compound is charged or neutral.

Figure S14 depicts the concentration-corrected P_m (P_m*C_{ext}) value that was found to be associated with the determined maximal $J_{pgp,active}$ of 1.6 x 10^{-4} µmol/cm^2/s (SA = 7.5) based on the magnitude of paracellular transport.

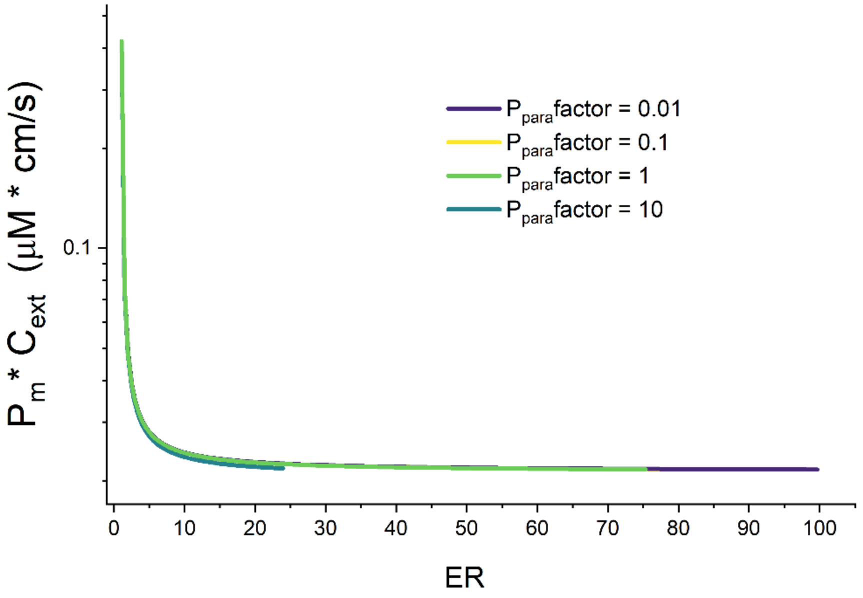

Figure S14: The P_m*C_{ext} threshold associated with a fixed maximal $J_{pgp,active}$ value of 1.6 x 10^{-4} µmol/cm^2/s as a function of ER values based on the magnitude of paracellular transport.
